# Oncogenic RAS commandeers amino acid sensing machinery to aberrantly activate mTORC1 in multiple myeloma

**DOI:** 10.1038/s41467-022-33142-x

**Published:** 2022-09-17

**Authors:** Yandan Yang, Arnold Bolomsky, Thomas Oellerich, Ping Chen, Michele Ceribelli, Björn Häupl, George W. Wright, James D. Phelan, Da Wei Huang, James W. Lord, Callie K. Van Winkle, Xin Yu, Jan Wisniewski, James Q. Wang, Frances A. Tosto, Erin Beck, Kelli Wilson, Crystal McKnight, Jameson Travers, Carleen Klumpp-Thomas, Grace A. Smith, Stefania Pittaluga, Irina Maric, Dickran Kazandjian, Craig J. Thomas, Ryan M. Young

**Affiliations:** 1 Lymphoid Malignancies Branch, Center for Cancer Research, National Cancer Institute, National Institutes of Health, Bethesda, MD 20892 USA; 2 Department of Medicine II, Heamatology/Oncology, Goethe University, 60323 Frankfurt, Germany; 3 Division of Preclinical Innovation, National Center for Advancing Translational Sciences, National Institutes of Health, Rockville, MD 20850 USA; 4 Biometric Research Branch, DCTD, National Cancer Institute, National Institutes of Health, Bethesda, MD 20892 USA; 5 Experimental Immunology Branch, National Cancer Institute, National Institutes of Health, Bethesda, MD 20892 USA; 6 Laboratory of Pathology, Center for Cancer Research, National Cancer Institute, National Institutes of Health, Bethesda, MD 20892 USA; 7 Hematology Service, Department of Laboratory Medicine, National Institutes of Health Clinical Center, Bethesda, MD 20892 USA; 8 Department of Medicine, University of Miami Health System, Miami, FL 33136 USA

**Keywords:** Myeloma, Cell signalling, Oncogenes

## Abstract

Oncogenic RAS mutations are common in multiple myeloma (MM), an incurable malignancy of plasma cells. However, the mechanisms of pathogenic RAS signaling in this disease remain enigmatic and difficult to inhibit therapeutically. We employ an unbiased proteogenomic approach to dissect RAS signaling in MM. We discover that mutant isoforms of RAS organize a signaling complex with the amino acid transporter, SLC3A2, and MTOR on endolysosomes, which directly activates mTORC1 by co-opting amino acid sensing pathways. MM tumors with high expression of mTORC1-dependent genes are more aggressive and enriched in RAS mutations, and we detect interactions between RAS and MTOR in MM patient tumors harboring mutant RAS isoforms. Inhibition of RAS-dependent mTORC1 activity synergizes with MEK and ERK inhibitors to quench pathogenic RAS signaling in MM cells. This study redefines the RAS pathway in MM and provides a mechanistic and rational basis to target this mode of RAS signaling.

## Abstract (web summary)

RAS mutations are commonly found in multiple myeloma (MM). Here, the authors show that oncogenic RAS mutations activate mTORC1 signalling in MM and combining mTORC1 and MEK/ERK inhibitors synergize to improve survival in preclinical models.

## Introduction

Multiple myeloma (MM) is the second most common hematological malignancy, accounting for nearly 35,000 new cancer cases a year within the United States (www.seer.cancer.gov). Substantial progress has been made treating this disease with the introduction of proteasome inhibitors and immunomodulatory drugs (IMiDs). These agents target vulnerabilities tied to the plasmacytic origins of MM and have significantly extended patient survival^1,2^. However, MM remains incurable and most patients will relapse and become refractory to existing treatments. Mutations targeting the RAS pathway are common in MM and associated with resistance to these therapies^3^. *KRAS* and *NRAS* are each mutated in about 20% of newly diagnosed MM cases^4,5^. MM is unusual in this regard, as other RAS-dependent tumor types typically rely on a single isoform of RAS^6^. RAS can signal through multiple effector pathways, perhaps most characteristically by activation of the classical MAP kinase (MAPK) pathway through RAF, MEK and ERK. Despite the high frequency of RAS mutations, the majority of MM tumors harboring RAS mutations have no detectable MEK activity by immunohistochemistry staining^7^ or analysis of MAPK-dependent transcription^8^, and MEK inhibitors have only had modest success treating MM patients in the clinic^9,10^. These findings suggest that RAS-dependent activation of the classical MAPK pathway is not the sole mode of RAS signaling in malignant plasma cells and point to an unidentified role for oncogenic RAS signaling in this disease.

To uncover mechanisms of pathogenic RAS signaling in MM, we implemented an unbiased proteogenomic pipeline that combined CRISPR-Cas9 screens to identify genes selectively essential in MM lines dependent on *KRAS* or *NRAS* expression, as well as quantitative mass spectrometry (MS) to determine protein interaction partners for mutant RAS isoforms in MM cells. This approach revealed the “essential interactome” of mutant RAS and highlighted the connection between RAS and SLC3A2. SLC3A2 (CD98, 4F2hc) is a component of several heterodimeric amino acid transporters for large neutral amino acids, including SLC3A2-SLC7A5, that serves to transport leucine and glutamine^11^. Proteomic analysis of RAS and SLC3A2 interaction partners and dependent signaling networks identified that mTORC1 was activated downstream of both RAS and SLC3A2. We determined that RAS coordinated the colocalization of SLC3A2 and MTOR on LAMP1 + endolysosomes, where RAS, SLC3A2 and MTOR cooperatively activated mTORC1. RAS accomplished this by subverting nutrient sensing pathways that normally regulate homeostasis through mTORC1. Inhibition of RAS-dependent mTORC1 activity enhanced reliance on MEK and ERK signaling in MM cells, and combinations of mTORC1 and MEK inhibitors resulted in a synthetic lethal phenotype that was profoundly toxic to RAS-dependent malignant cells. Thus, our work details a previously uncharacterized concept in pathogenic RAS signaling and outlines potential therapeutic opportunities to exploit this signaling mechanism.

## Results

### Proteogenomic screens in MM

**Fig. 1:**
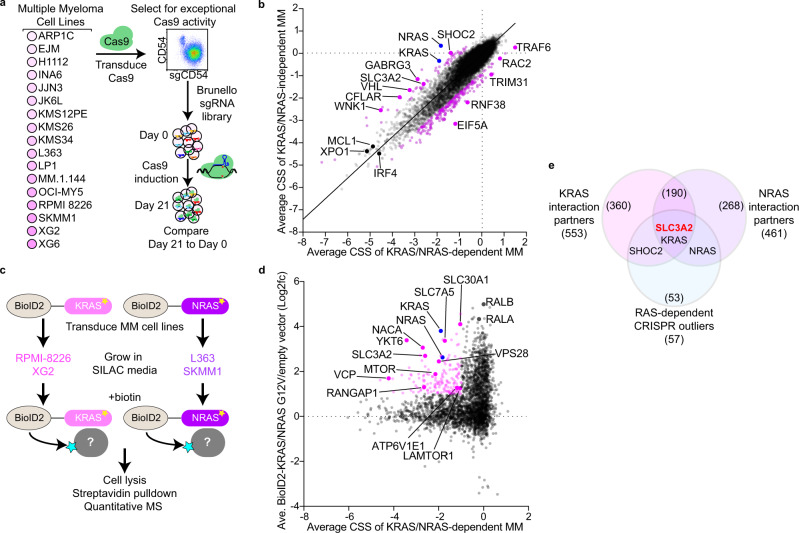
Proteogenomic screens reveal a relationship between SLC3A2 and RAS in multiple myeloma. **a** Workflow for CRISPR screens in MM cell lines. **b** Scatter plot of the average CRISPR screen scores (CSS) for RAS-dependent MM lines (x-axis) vs. RAS-independent MM lines (*y*-axis). Outliers were determined by an extra sum-of-squares *F* test (*P* < 0.05) and are labeled in purple and blue. **c** Workflow of BioID2-based mutant KRAS and NRAS proximity labeling SILAC mass spectrometry (MS) studies. **d** Essential interactome of G12V RAS isoforms in RAS-dependent MM. Average CSS for KRAS/NRAS-dependent MM cell lines (*x*-axis) plotted by average combined enrichment of BioID2-KRAS^G12V^/ BioID2-NRAS^G12V^ relative to empty vector. Essential interactome (≤−1.0 CSS and ≥1.0 log2fc BioID2-RAS) is labeled in pink. **e** Venn diagram of protein interaction partners substantially enriched (≥2.0 log2fc) in BioID2-KRAS^G12V^ and BioID2-NRAS^G12V^ with RAS-dependent outlier genes identified in Fig. 1b. Source data are provided as a Source Data file.

We conducted CRISPR-Cas9 screens to identify genes essential to malignant growth and survival in 17 MM cell lines (Fig. 1a). To maximize the sensitivity and utility of these CRISPR screens, Cas9-engineered MM lines were first selected for exceptional exonuclease activity as determined by reduction in CD54 levels following expression of a CD54-targeted single guide RNA (sgRNA) (Fig. 1a). Cas9 clones with high knockout efficiency were subsequently screened with the third-generation genome-wide Brunello sgRNA library^12^ to identify essential genes after 21 days of growth. For each gene we determined the CRISPR screen score (CSS), a metric of how deletion of a gene affects cell growth and survival, akin to a Z-score^13^. Deletion of genes known to be essential to MM biology, including *XPO1*^14^, *IRF4*^15^, *MYC*^16^ and *MCL1*^17^ were toxic to all MM lines and had negative CSS values (Fig. S1a). In contrast, *FAM46C* and *ID2* acted as tumor suppressors in many MM lines as indicated by their positive CSS values (Fig. S1a), consistent with previous results^18,19^. Comparison of our CRISPR dataset to the 20 MM cell lines within DepMap^20^ found general agreement (Fig. S1b), although our approach identified additional MM-specific essential genes, likely due to the superior performance of the Brunello library^21^.

We next parsed the CRISPR screen data to identify genes preferentially essential in RAS-dependent MM. Our screens identified eight MM cell lines reliant on *KRAS* or *NRAS* expression for their growth and survival (Fig. S1c, d). All RAS-dependent MM lines harbored oncogenic RAS mutations, with the exception of KMS26 which expressed wild type *KRAS*. In contrast, EJM, JJN3, JK6L and XG6 expressed mutant isoforms of RAS but were not sensitive to *KRAS* or *NRAS* knockout. We compared CRISPR screen results from RAS-dependent MM lines (grouped *KRAS*-dependent and *NRAS*-dependent, x-axis) to MM lines insensitive to *KRAS* or *NRAS* deletion (*y*-axis) in Fig. 1b. Outlier genes selectively more toxic in RAS-dependent or RAS-independent MM lines were identified by an extra sum-of-squares *F* test (*p* < 0.05) (Fig. 1b). In addition to *NRAS* and *KRAS* themselves, the RAS-dependent outliers included *SHOC2*, which was previously shown to activate MAP kinase (MAPK) signaling downstream of oncogenic RAS^22^. However, most other RAS-dependent outliers have no reported link to RAS signaling, and pathway analysis of these genes yielded no significant enrichments or clues to their function.

To unlock additional insight from these CRISPR screens, we employed an orthogonal proteomic approach to identify proteins that interact with RAS isoforms in MM cells. BioID2 is a promiscuous biotin ligase that can biotinylate proteins within a 10–30 nm distance^23^, and we ectopically expressed BioID2 fused to KRAS^G12V^ in RPMI 8226 and XG2, or NRAS^G12V^ in SKMM1 and L363 MM cells (Fig. 1c). Biotinylated proteins were purified from these cells by streptavidin pulldown and enumerated using quantitative stable isotope labeling by amino acids in cell culture mass spectrometry (SILAC-MS) (Fig. 1c). These experiments identified numerous proteins enriched relative to empty vector (pBMN-Lyt2-BioID2) expressing BioID2 alone (Fig. S2a, b), including many known RAS effectors (Fig. S2c). To focus on the RAS interactors most essential to growth and survival in MM, we compared the enrichment of proteins within BioID2-RAS interactomes to the CRISPR screen data averaged for all RAS-dependent MM cells (Fig. 1d). Notably, these essential interactomes did not include classical RAS effectors––such as BRAF and RALA – because these genes were not determined to be essential by CRISPR screening, although it remains possible this is due to redundancy among paralogous genes. Regardless, this essential interactome highlighted associations between RAS and MTOR, several solute-carrier (SLC) genes, and many cellular trafficking proteins. Shared KRAS and NRAS interaction partners were significantly enriched in Gene Ontology (GO) pathway gene sets^24^ associated with membrane and vesicular trafficking (Fig. S2d). We next compared KRAS and NRAS protein interaction partners (≥2 log2fc) with the set of genes found to be significantly more essential within RAS-dependent tumors (Fig. 1b). Remarkably, only SLC3A2 interacted with both KRAS and NRAS, and was more selectively essential in RAS-dependent MM lines (Fig. 1e).

### SLC3A2 regulates mTORC1 signaling in RAS-dependent MM cells

**Fig. 2:**
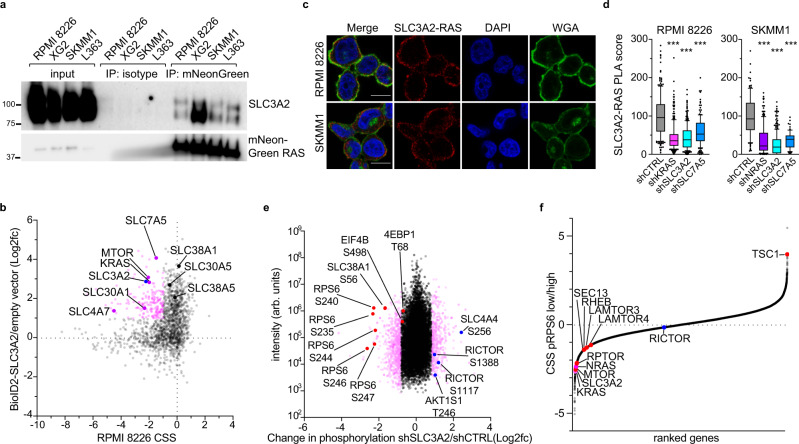
SLC3A2 associates with RAS and controls mTORC1 activity. **a** Co-immunoprecipitation of SLC3A2 with mutant isoforms of mNeonGreen-tagged KRAS and NRAS. mNeonGreen-tagged KRAS^G12D^ was used in RPMI 8226 and XG2, NRAS^G12D^ in SKMM1 and NRAS^Q61L^ in L363. Representative blots; *n* = 4. **b** Essential interactome of SLC3A2 in RPMI 8226. CRISPR screen score (CSS; x-axis) plotted by the BioID2-SLC3A2/empty vector enrichment (y-axis). Essential interactome is labeled in pink (≤−1.0 CSS and ≥1.0 log2fc BioID2-SLC3A2). **c** Proximity ligation assay (PLA) (red) of SLC3A2 and RAS in RPMI 8226 and SKMM1 cells. Cells were counterstained with DAPI (blue) and wheat germ agglutinin (WGA; green). Scale bar is 10 μm, representative images; *n* = 3. **d** PLA score of cells transduced with indicated shRNAs following puromycin selection for two days. Data from three independent experiments for KRAS, NRAS and SLC3A2 knockdown, and 2 independent experiments for SLC7A5 knockdown. Data pooled from independent experiments; the number of cells quantified per condition is listed in the source data file. *** denotes *P* value < 0.0001 by one-way ANOVA with Dunnett’s post test; box plots represent median and 25–75% of data, whiskers incorporate 10–90% of data, and outliers are displayed as dots. **e** Global changes in phosphorylation measured by quantitative MS following knockdown of SLC3A2 in SKMM1. Change in phosphorylation (log2fc) in cells expressing shSLC3A2 vs. shCTRL (*x*-axis) plotted by the measured intensity (*y*-axis). **f** Identification of genes that regulate phosphorylation of RPS6 at S240/244 by CRISPR screening. Averaged CRISPR screen results from sorted RPMI 8226 and SKMM1 cells comparing cells with low p-RPS6 vs. high p-RPS signal. All values are averaged for both cell lines except for KRAS and NRAS, which are shown for RPMI 8226 and SKMM1, respectively. Source data are provided as a Source Data file.

SLC3A2 (CD98, 4F2hc) is integral to amino acid transport in cells^11^, and high levels of SLC3A2 protein expression are correlated to aggressive MM^25^. We confirmed that SLC3A2 associated with RAS isoforms in MM cells by co-immunoprecipitation with ectopically expressed mutant isoforms of KRAS or NRAS in RAS-dependent MM cell lines (Fig. 2a). To explore SLC3A2 function in MM cells, we expressed a BioID2-SLC3A2 construct in RPMI 8226 to determine SLC3A2 protein interaction partners by MS. We resolved the SLC3A2 essential interactome by plotting the BioID2-SLC3A2 protein enrichment (y-axis) against CSS values for each gene in RPMI 8226 (x-axis) (Fig. 2b). As expected, we observed a strong interaction between KRAS and SLC3A2 in RPMI 8226 cells. In addition, we detected robust interactions with other essential and non-essential SLC-family members, including SLC7A5, SLC38A1, SLC30A5 and SLC4A7. SLC3A2 is known to heterodimerize with SLC7A5^26^, but many of these SLC proteins have not been previously described to interact with SLC3A2 and it is unclear if these associations represented novel heterodimers with SLC3A2 or reflected the proximity of these proteins in the cell membrane. However, our BioID2 data suggested that SLC3A2 primarily pairs with SLC7A5, which would form a leucine and glutamine transporter^26^.

We next used the proximity ligation assay (PLA), which can quantitatively visualize protein-protein interactions within tens of nanometers as discrete puncta in situ^27^, to study interactions between endogenous SLC3A2 and RAS in the KRAS-dependent RPMI 8226 and NRAS-dependent SKMM1 MM cells. We observed numerous bright PLA puncta confirming the proximity of SLC3A2 and RAS in both MM lines (Fig. 2c; red). We found PLA signal near the plasma membrane, stained by wheat germ agglutinin (WGA; green), as well as within the cytosol. Immunofluorescence staining of SLC3A2 and RAS showed that both proteins are predominantly localized to the plasma membrane but also share a diffuse staining throughout the cytoplasm that was highlighted by the PLA (Fig. S3a). Knockdown of RAS isoforms or SLC3A2 abrogated SLC3A2-RAS PLA signal, demonstrating the specificity of detecting this interaction by PLA (Fig. 2d). Knockdown of SLC7A5 also abolished PLA signal between SLC3A2 and RAS (Fig. 2d), providing more evidence that SLC3A2 likely interacts with RAS as part of a heterodimer with SLC7A5, as supported by our proteomic data (Fig. 2b). Finally, analysis of SLC3A2-RAS PLA in the cohort of MM lines used for CRISPR screening found that RAS-dependent MM cell lines had significantly more PLA puncta per cell than RAS-independent MM cell lines (Fig. S3b).

We probed the role of SLC3A2 in MM signaling by enumerating changes in global phosphorylation by quantitative MS following SLC3A2 knockdown in SKMM1 cells (Fig. 2e, Fig. S3c). SLC3A2 knockdown substantially decreased phosphorylation of RPS6 at multiple serine residues. RPS6 is a target of p70S6K, a known effector downstream of mTORC1^28^. MTOR regulates cellular growth, metabolism and proliferation as a member of two multicomponent signaling complexes, mTORC1 and mTORC2. mTORC1 signaling is gated by the availability of nutrients, including amino acids, and SLC3A2 has been previously implicated in mTORC1 regulation in its role as an amino acid transporter^26^. Western blot analysis following SLC3A2 knockdown in RAS-dependent MM lines found significant reductions in mTORC1 targets (p-p70S6K (T389) and p-4EBP1 (S65)) but minimal changes in the mTORC2 target PKCα (p-T638/641) (Fig. S3d, e). Of note, phosphorylation of MEK, a target of RAS signaling, was only modestly reduced by SLC3A2 knockdown, suggesting that although SLC3A2 and RAS are interaction partners, SLC3A2 does not substantially control MEK signaling downstream of RAS (Fig. S3d).

We next used phospho-RPS6 staining as an endpoint for whole-genome CRISPR screens to identify genes essential to mTORC1 activity in RAS-dependent MM cells in an unbiased fashion. The experimental design is shown in Figure S3f. RPMI 8226 and SKMM1 cells expressing Cas9 were transduced with the Brunello library and allowed to grow for 10 days to permit gene knockout. At this point an input sample was reserved and the remaining cells were fixed, stained and FACS sorted to isolate cells with either high or low anti-phospho-S240/S244-RPS6 signal. We compared the enrichment of sgRNAs from the p-RPS6 low cells to the p-RPS6 high cells (Fig. 2f). Cells expressing sgRNAs targeting *SLC3A2*, *MTOR*, *RPTOR*––a component of mTORC1––and oncogenic RAS (*KRAS* in RPMI 8226 and *NRAS* in SKMM1 are shown in pink) were highly enriched in cells with low p-RPS6 staining (Fig. 2f), demonstrating that these genes were essential to mTORC1 activity in these cells. Likewise, deletion of regulators of the amino acid sensing machinery (*SEC13*, *LAMTOR3*, *LAMTOR4*) and *RHEB* reduced p-RPS6 signal, whereas knockout of a negative regulator of mTORC1, *TSC1*, increased p-RPS6 staining. Cells expressing sgRNAs targeting the mTORC2-specific component, RICTOR, were not enriched in either population. Taken together, these data confirmed that SLC3A2 regulated mTORC1 signaling in MM and indicated that oncogenic RAS was also required for mTORC1 activity.

### RAS controls association of SLC3A2 with MTOR on LAMP1+ endolysosomes

**Fig. 3:**
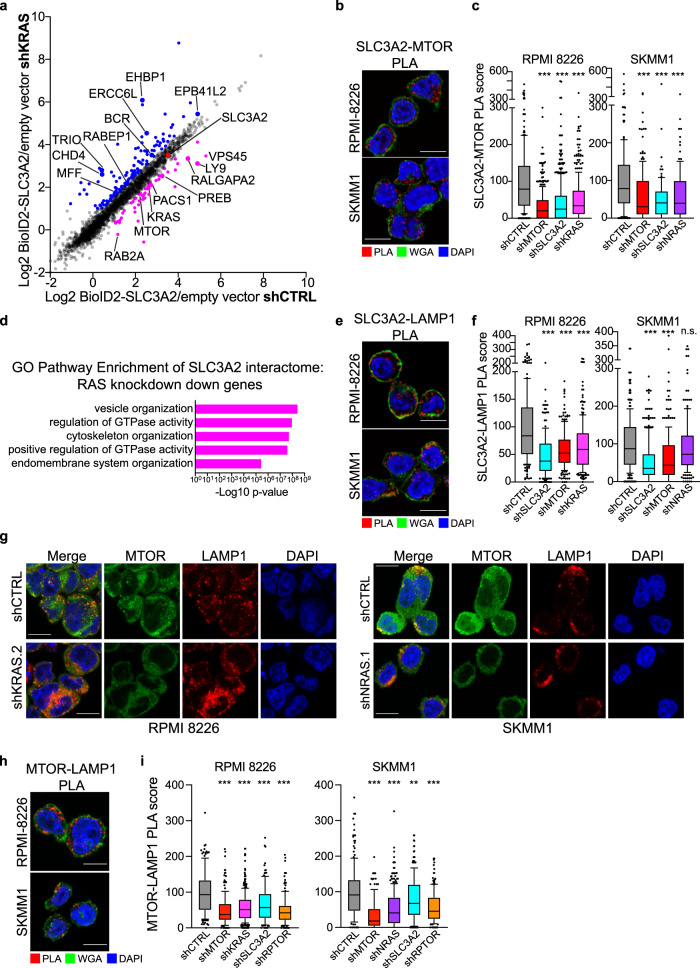
RAS regulates localization of SLC3A2 and MTOR to endolysosomes. **a** BioID2-SLC3A2 interactomes in XG2 cells transduced with control shRNA (x-axis) or shKRAS (y-axis), proteins are colored that decreased by at least 0.5 log2fc (purple) or increased by at least 0.5 log2fc (blue). **b** Proximity ligation assay (PLA) (red) of SLC3A2 with MTOR in RPMI 8226 and SKMM1 cells. Cells counterstained with DAPI (blue) and wheat germ agglutinin (WGA; green); Scale bar is 10 μm. Representative images; *n* = 3. **c** SLC3A2-MTOR PLA score of cells transduced with indicated shRNAs. Details about box plots below. **d** Gene Ontology pathway enrichment of SLC3A2 interactors that decreased by at least 0.5 log2fc following RAS knockdown. Bonferroni corrected *P* value plotted on the x-axis. **e** PLA of SLC3A2 with LAMP1 (red) in RPMI 8226 and SKMM1 cells. Cells counterstained with DAPI (blue) and WGA (green); Scale bar is 10 μm. Representative images; *n* = 3. **f** SLC3A2-LAMP1 PLA score of cells transduced with control shRNA or shRNAs specific for KRAS, NRAS, SLC3A2 and MTOR. Details about box plots below. **g** Immunofluorescence images of MTOR (green), LAMP1 (red) and DAPI (blue) in RPMI 8226 and SKMM1 cells expressing shCTRL, shKRAS.2 or shNRAS.1. Scale bar is 10 μm. Representative images; *n* = 3. **h** PLA of MTOR with LAMP1 (red) in RPMI 8226 and SKMM1 cells. Cells counterstained with DAPI (blue) and WGA (green); Scale bar is 10 μm. Representative images; *n* = 3. **i** MTOR-LAMP1 PLA score of cells transduced with control shRNA or shRNAs specific for MTOR, KRAS, NRAS, SLC3A2 and RPTOR. Details about box plots below. For all box plots: Data are pooled independent experiments; the number of cells quantified per condition indicated in the source data file. *** denotes *P* value <0.0001, ** denotes *P* value = 0.0023, n.s. denotes not significant, as determined by one-way ANOVA with Dunnett’s post test. The number of independent experiments and the number of cells quantified for each condition can be found in the source data. Box plots represent median and 25–75% of data, whiskers incorporate 10–90% of data, outliers are displayed as dots. Source data are provided as a Source Data file.

To understand how RAS may cooperate with SLC3A2 in MM cells, we evaluated changes in SLC3A2 protein interaction partners following RAS knockdown. We expressed BioID2-SLC3A2 in four RAS-dependent MM lines and enumerated changes in protein interactions by SILAC MS two days after induction of either a control shRNA or shRNAs targeting KRAS or NRAS, corresponding to the mutant isoform of RAS expressed in each MM cell line. Data for the KRAS-dependent MM line XG2 is shown in Fig. 3a, and protein interactions that either decreased or increased by at least 0.5 log2fc are depicted in purple or blue, respectively, with outliers labeled if they were found in two or more MM lines. We found that SLC3A2 association with MTOR consistently decreased following RAS knockdown (Fig. 3a). We next used PLA to visualize interactions between endogenous MTOR and SLC3A2, which generated bright puncta throughout the cytosol (Fig. 3b, red). The MTOR-SLC3A2 PLA was specific since knockdown of its constituent parts, MTOR and SLC3A2, nearly eliminated PLA signal (Fig. 3c). In addition, knockdown of KRAS or NRAS substantially reduced associations between MTOR and SLC3A2, confirming the BioID2 results in Fig. 3a and demonstrating that RAS governs associations between SLC3A2 with MTOR.

To gain further insight into the mechanisms by which RAS regulates SLC3A2 and MTOR associations, we performed GO pathway analysis on BioID2-SLC3A2 interactors that were reduced by at least 0.5 log2fc following RAS knockdown in any of the four MM lines tested. These proteins were enriched in pathways associated with vesicle and endomembrane organization (Fig. 3d), suggesting that RAS may control localization of SLC3A2 to endomembranes. SLC3A2-SLC7A5 has been previously characterized on lysosomal membranes, where it promoted entry of leucine into the lysosomal lumen to stimulate V-ATPase and mTORC1 activity^29^. To test if RAS regulated localization of SLC3A2 to endolysosomes, we developed a PLA pair between SLC3A2 and a marker of endolysosomes, LAMP1. SLC3A2-LAMP1 PLA puncta were observed throughout the cytosol in RPMI 8226 and SKMM1 MM cells (Fig. 3e, red). This interaction was specific since knockdown of SLC3A2 significantly disrupted SLC3A2-LAMP1 PLA signal (Fig. 3f). However, RAS knockdown only significantly decreased the number of SLC3A2-LAMP1 PLA puncta in RPMI 8226 (Fig. 3f). Accordingly, RAS knockdown did not meaningfully change surface expression of SLC3A2 in MM lines (Fig. S3g), indicating that regulation of SLC3A2 intracellular trafficking by RAS may be more nuanced. Notably, MTOR expression was also required to localize SLC3A2 to LAMP1 (Fig. 3f), suggesting that RAS may control SLC3A2 trafficking via regulation of MTOR.

mTORC1 activity can be regulated by recruitment of MTOR to endolysosomes by the Ragulator-Rag complex^30^. We hypothesized that RAS could control localization of MTOR to endolysosomal membranes to bring SLC3A2 and MTOR together. Immunofluorescence of MTOR and LAMP1 in RPMI 8226 and SKMM1 revealed that MTOR formed foci throughout the cytosol which overlapped with LAMP1 staining (Fig. 3g), consistent with MTOR engaged in chronic mTORC1 signaling. Knockdown of KRAS or NRAS substantially diminished these MTOR foci, although LAMP1 staining was not reduced (Fig. 3g), signifying that oncogenic RAS regulated the recruitment of MTOR to the mTORC1 complex. These data suggested that RAS-dependent regulation of MTOR localization to endolysosomes could be a mechanism by which RAS indirectly coordinated SLC3A2 subcellular localization.

We wondered whether SLC3A2 also controlled localization of MTOR to endolysosomes, since disruption of SLC3A2 expression reduced mTORC1 activity (Fig. 2e, Fig. S3d). While SLC3A2 knockdown did not reduce LAMP1 staining in MM cells (Fig. S3h), changes to MTOR colocalization with LAMP1 were difficult to discern by immunofluorescence. To quantify association of MTOR and LAMP1 we developed an MTOR-LAMP1 PLA pair (Fig. 3h). Knockdown of MTOR substantially reduced PLA signal, demonstrating its specificity (Fig. 3i), and RAS knockdown greatly reduced MTOR association with LAMP1 (Fig. 3i), in accord with the immunofluorescence data (Fig. 3g). RPTOR has been reported to regulate MTOR localization to mTORC1^31^, and RPTOR knockdown nearly abolished associations between MTOR and LAMP1. Finally, we found that SLC3A2 expression was required for MTOR to colocalize with LAMP1 (Fig. 3i), suggesting that expression of RAS, SLC3A2 and MTOR were all necessary for these proteins to reside on endolysosomal membranes.

### mTORC1 activity is a predominant feature of RAS signaling in MM

**Fig. 4:**
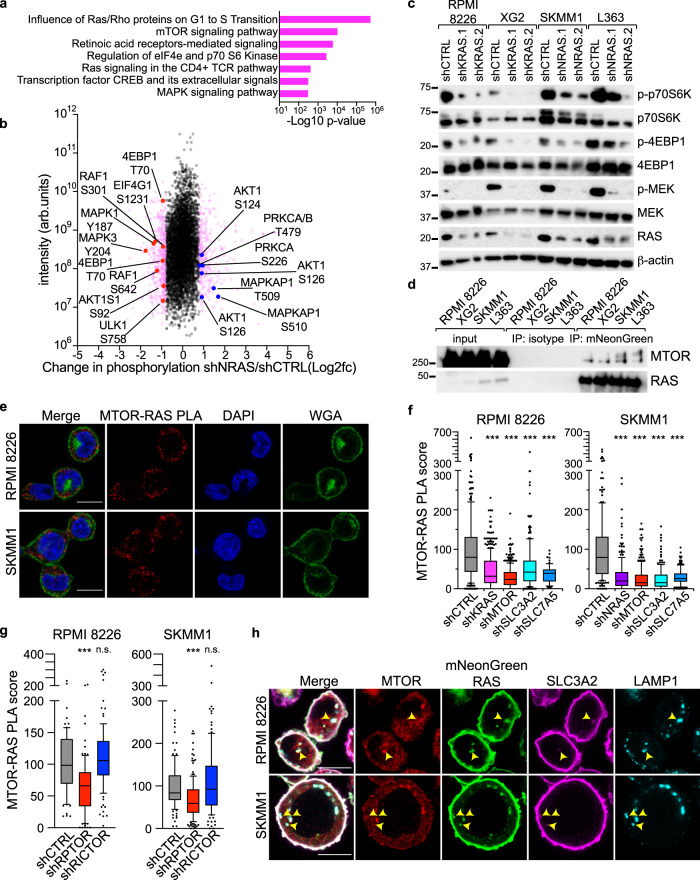
RAS controls mTORC1 activity in multiple myeloma. **a** Pathway analysis of proteins with ±0.8 log2 fold changes in phosphorylation in SKMM1 cells transduced with shNRAS compared to control shRNA as determined by quantitative mass spectrometry. **b** Scatter plot of changes in phosphorylation following NRAS knockdown (shNRAS/shCTRL; x-axis) vs. intensity (y-axis). Proteins in the MTOR and MAPK signaling pathways are labeled. **c** Western blot analysis of mTORC1 and MEK signaling following KRAS or NRAS knockdown in the indicated MM lines. Representative blots, *n* = 5. **d** Co-immunoprecipitation of MTOR with mutant isoforms of mNeonGreen-tagged KRAS and NRAS. KRAS^G12D^ was used in RPMI 8226 and XG2, NRAS^G12D^ in SKMM1 and NRAS^Q61L^ in L363. Representative blots; *n* = 3. **e** Proximity ligation assay (PLA) of MTOR-RAS (red) in RPMI 8226 and SKMM1 cells. Cells counterstained with DAPI (blue) and wheat germ agglutinin (WGA; green); Scale bar is 10 μm. Representative images; *n* = 3. **f** MTOR-RAS PLA score of cells transduced with control shRNA or shRNAs specific for KRAS (*n* = 3), NRAS (*n* = 3), SLC3A2 (*n* = 3), SLC7A5 (*n* = 2) and MTOR (*n* = 3). Data pooled from independent experiments; the number of cells quantified per condition listed in the source data file. *** denotes *P* value <0.0001 by one-way ANOVA with Dunnett’s post test. Box plots represent median and 25–75% of data, whiskers incorporate 10–90% of data, outliers are displayed as dots. **g** MTOR-RAS PLA in RPMI 8226 and SKMM1 cells expressing shCTRL (*n* = 3), shRPTOR (*n* = 3) or shRICTOR (*n* = 3) shRNAs. Data pooled from independent experiments; the number of cells quantified per condition listed in the source data file. *** denotes *P* value <0.0001, n.s. denotes not significant, by one-way ANOVA with Dunnett’s post test. Box plots represent median and 25–75% of data, whiskers incorporate 10–90% of data, outliers are displayed as dots. **h** Immunofluorescence of MTOR (red), ectopically expressed mNeonGreen-KRAS^G12D^ in RPMI 8226 or mNeonGreen-NRAS^G12D^ SKMM1 cells (green), SLC3A2 (magenta) and LAMP1 (cyan). Yellow arrows highlight areas of overlap. Scale bar is 10 μm. Representative images; *n* = 3. Source data are provided as a Source Data file.

Our data suggested that oncogenic RAS regulated mTORC1 signaling in MM, but it was unclear whether this was a dominant feature of RAS signaling in this disease. To obtain an unbiased view of RAS signaling in a malignant plasma cell, we employed quantitative MS to enumerate changes in global phosphorylation following knockdown of NRAS in SKMM1 cells (Fig. S4a). Pathway enrichment of proteins whose phosphorylation changed ±0.8 log2fc identified that the mTORC1 pathway and, to a lesser extent, the MAPK pathway, are the prominent RAS effector pathways in SKMM1 cells (Fig. 4a). NRAS knockdown markedly decreased phosphorylation on targets of mTORC1 signaling (4EBP1, EIF4G1, ULK1) and MAPK signaling (RAF1, MAPK1, MAPK3). In contrast, NRAS knockdown resulted in increased phosphorylation of mTORC2 components and its downstream signaling effectors (MAPKAP1, AKT1, PRKCA) (Fig. 4b), perhaps due to compensatory signaling feedback between mTORC1 and mTORC2. We confirmed these proteomic findings by western blot analysis in additional RAS-dependent MM cell lines. KRAS or NRAS knockdown decreased phosphorylation of mTORC1 targets, p70S6K (T389) and 4EBP1 (S65), in all RAS-dependent MM lines tested (Fig. 4c). We observed little or no change in mTORC1 signaling upon RAS knockdown in MM cells not dependent on RAS expression (Fig. S4b). Moreover, disruption of RAS expression also reduced phosphorylation of MEK (S217/221) in these MM lines (Fig. 4c), consistent with the MS phosphoproteomic analysis.

Our BioID2 studies identified that RAS strongly interacted with MTOR (Fig. 1d), suggesting that RAS may regulate mTORC1 signaling as part of a complex with MTOR. We confirmed that MTOR associated with RAS isoforms in MM cells by co-immunoprecipitation with ectopically expressed mutant isoforms of KRAS or NRAS in four MM cell lines (Fig. 4d). We next established that endogenous MTOR and RAS associated in RPMI 8226 and SKMM1 MM lines by PLA (Fig. 4e, red). Knockdown of MTOR and RAS isoforms quenched PLA signals and confirmed the specificity of this PLA pair (Fig. 4f). Additionally, we found that MTOR-RAS PLA signal was significantly enriched in RAS-dependent versus RAS-independent MM cell lines (Fig. S4c). Remarkably, SLC3A2 and SLC7A5 knockdown abolished MTOR-RAS PLA signal (Fig. 4f), suggesting that RAS can only interact with MTOR in the presence of SLC3A2-SLC7A5. MTOR-RAS PLA signal was also significantly correlated to both SLC3A2-RAS PLA signal (Fig. S4d) and the SLC3A2 CSS (Fig. S4e), further linking SLC3A2 with MTOR-RAS associations.

A previous study described that oncogenic RAS isoforms directly bound and activated mTORC2 in melanoma and other solid tumor cell lines^32^. To test the role of mTORC1 and mTORC2 in RAS-dependent MM, we assessed whether associations between RAS and MTOR were altered by knockdown of RPTOR or RICTOR, components specific to either mTORC1 or mTORC2, respectively. We found that knockdown of RPTOR substantially decreased the number of MTOR-RAS PLA puncta in RPMI 8226 and SKMM1 cells (Fig. 4g). In contrast, RICTOR knockdown had no effect on MTOR-RAS PLA (Fig. 4g). Accordingly, we co-immunoprecipitated RPTOR but not RICTOR in most RAS-dependent MM cell lines that ectopically expressed mutant RAS (Fig. S5a). We also observed robust interactions between endogenously expressed RPTOR and RAS in MM cells by PLA but failed to find associations between RICTOR and RAS (Fig. S5b). These data confirm that RAS associates with mTORC1 within MM cells and established that mTORC1 signaling is a central feature of RAS and SLC3A2-dependent signaling in MM.

MTOR-RAS PLA puncta were cytosolic and generally not coincident with the plasma membrane (Fig. 4e), consistent with localization to mTORC1 complexes on the surface of endolysosomes. These data support a model in which oncogenic RAS signals from endolysosomal membranes, yet oncogenic KRAS and NRAS are thought to chiefly signal from the plasma membrane. However, we found that a fraction of ectopically expressed mutant KRAS or NRAS fused to mNeonGreen colocalized with endolysosomes stained with Lysotracker Red in RPMI 8226 and SKMM1 cells (Fig. S5c), demonstrating that RAS colocalized with endolysosomes in MM cells. Moreover, we found that endogenous KRAS localized to LAMP1 + endolysosomes by both immunofluorescence (Fig. S5d) and PLA (Fig. S5e) in RPMI 8226, although we were unable to satisfactorily visualize colocalization with pan-RAS or NRAS-specific antibodies. KRAS-LAMP1 PLA signal was dependent upon expression of SLC3A2 and components of mTORC1, MTOR and RPTOR (Fig. S5f), suggesting that oncogenic RAS only localized to endolysosomes in complex with SLC3A2 and mTORC1. Indeed, we observed that RAS, MTOR and SLC3A2 colocalized with LAMP1 in RPMI 8226 and SKMM1 MM cells ectopically expressing fluorescent fusion constructs of KRAS^G12D^ or NRAS^G12D^ (Fig. 4h, yellow arrows), demonstrating that SLC3A2, RAS and MTOR form a complex on LAMP1 + vesicles within MM cells.

We next sought to understand the role of RAS activity in regulating molecular associations between SLC3A2, MTOR and RAS. RPMI 8226 and SKMM1 cells were transduced with either constitutively active (G12D) or dominant negative (S17N) versions of KRAS or NRAS, respectively. We then evaluated these cells by PLA to measure associations between RAS, MTOR and SLC3A2. We found that expression of dominant negative RAS substantially reduced MTOR-RAS and SLC3A2-MTOR PLA signal in both MM lines (Fig. S5g), suggesting that these protein associations are dependent on RAS activity. In contrast, we observed variable changes on SLC3A2-RAS PLA in MM cells expressing constitutively active or dominant negative RAS isoforms (Fig. S5g), and we cannot conclude if RAS activity is necessary for the association of RAS with SLC3A2. However, these data are consistent with a requirement of RAS activity for localization of RAS, SLC3A2 and MTOR to mTORC1 complexes on endolysosomes.

### Oncogenic RAS co-opts amino acid sensing to activate mTORC1

**Fig. 5:**
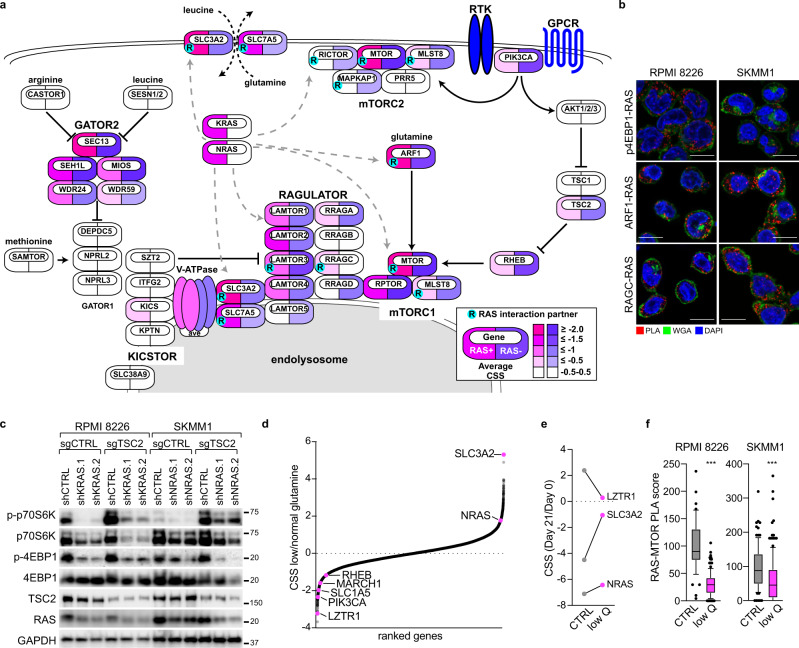
Oncogenic RAS activates mTORC1 by co-opting the amino acid sensing machinery. **a** Pathway diagram of MTOR signaling. Symbols are colored by the average CRISPR screen score (CSS) in RAS-dependent (pink) and RAS-independent (purple) MM cell lines and marked with a cyan dot containing an “R” if they were found to interact with mutant KRAS and NRAS in BioID2 experiments (≥2.0 log2fc). **b** Indicated proximity ligation assays (PLA) in RPMI 8226 and SKMM1 cells with PLA (red), wheat germ agglutinin (WGA; green) and DAPI (blue). Scale bar is 10 μm. Representative images; *n* = 2. **c** Western blot analysis of mTORC1 signaling in RPMI 8226 and SKMM1 cells following KRAS or NRAS knockdown and expression of either control (sgCTRL) or TSC2 sgRNAs. Representative blots; *n* = 3. **d** CRISPR modifier screen results identify genes with differential essentialities under glutamine restriction vs. normal glutamine conditions in SKMM1. CSS of genes from cells grown under glutamine restriction vs. normal glutamine conditions on *y*-axis. **e** Change in CSS for LZTR1, SLC3A2 and NRAS from Day 21 vs. Day 0 for normal glutamine conditions (gray) and under glutamine restriction (pink). **f** MTOR-RAS Proximity ligation assay (PLA) scores for cells under normal glutamine conditions (gray; *n* = 3) and under acute (12 h) glutamine restriction (pink; *n* = 3). Data pooled from independent experiments; the number of cells quantified per condition indicated in the source data file. *** denotes *P* value <0.0001 determined by Mann–Whitney unpaired two-tailed *t*-test. Box plots represent median and 25–75% of data, whiskers incorporate 10–90% of data, outliers are displayed as dots. Source data are provided as a Source Data file.

Our proteogenomic screens illustrated a profound connection between RAS and MTOR signaling in MM. A schematic of MTOR signaling is shown in Fig. 5a with individual components shaded by their average CSS for RAS-dependent (pink) and RAS-independent (purple) cell lines. Proteins enriched in oncogenic KRAS and NRAS BioID2 experiments by an average of ≥2 log2fc over empty vector are marked by a cyan circle with an ‘R’. This map shows interactions between RAS, SLC3A2, SLC7A5 and MTOR that we have characterized above. Interestingly, genes that comprise mTORC1 were highly essential in all MM cells yet components of mTORC2 were only necessary in RAS-independent MM cell lines, suggesting that RAS-independent cells rely on upstream growth factor or chemokine receptor signaling to stimulate phosphoinositide 3-kinase (PI3-K). Nonetheless, we found that oncogenic RAS strongly interacted with components of mTORC2 in BioID2 experiments, although we were unable to confirm associations between RAS and RICTOR by immunoprecipitation or PLA (Fig. S5a, b). These data raise the possibility that RAS may act to suppress mTORC2 signaling in MM, in accord with changes to global protein phosphorylation following RAS knockdown (Fig. 4b). In addition, Fig. 5a shows that RAS associated with multiple components of mTORC1 signaling in BioID2 experiments, including LAMTOR3, RRAGC and ARF1. We used PLA to visualize interactions of RAS with RAGC, a component of the Ragulator, and ARF1, which can act as a glutamine sensor to activate mTORC1^33^ (Fig. 5b). Moreover, we detected RAS in close association with p-S65-4EBP1 by PLA, suggesting that RAS was present at the site of active mTORC1 signaling (Fig. 5b).

Activation of mTORC1 requires concomitant engagement of nutrient sensing machinery and inhibition of TSC2 to permit RHEB association with mTORC1. TSC2 inhibition can be achieved downstream of PI3-K^34^ or ERK^35^. RAS has been previously described to directly bind and activate isoforms of PI3-K in solid cancers^36^ and RAS can activate classical MAPK signaling upstream of ERK in MM (Fig. 4a–c). To test if RAS is primarily regulating mTORC1 via TSC2 in MM, we examined the effect of RAS knockdown in MM cells lacking TSC2 expression. RPMI 8226 and SKMM1 cells were first transduced with control or TSC2 sgRNAs, followed by transduction with control or RAS shRNAs. We found that TSC2 deletion markedly increased phosphorylation of 4EBP1 (S65) and p70S6K (T389), yet even these elevated levels of phosphorylation were still dependent on RAS expression (Fig. 5c). These data demonstrated that RAS could regulate mTORC1 signaling through mechanisms separate from TSC2.

Our data suggested the amino acid transporter activity of SLC3A2 was required for RAS-dependent activation of mTORC1. SLC3A2-SLC7A5 must antiport glutamine in order to transport large neutral amino acids, such as leucine, isoleucine, phenylalanine and valine, into the cell^26^. We reasoned that glutamine restriction would inhibit the transporter activity of SLC3A2-SLC7A5, and we used this assumption to test the contribution of SLC3A2-SLC7A5 transporter activity to gene essentialities in a CRISPR modifier screen. The experimental setup is shown in Figure S6a. Briefly, SKMM1 cells expressing Cas9 were transduced with the Brunello library and allowed to grow for one week under normal growth conditions. At this point, cells were split into two pools that were grown either in normal glutamine conditions (0.3 g/L) or under glutamine restriction (0.03 g/L) for two additional weeks. We then determined differential gene essentialities in low versus normal glutamine conditions (Fig. 5d), or in each condition versus Day 0 (Fig. 5e). We found that *SLC3A2* became substantially less essential under glutamine restriction (Fig. 5d, e), demonstrating that the amino acid transporter function of SLC3A2 was required under normal conditions. Likewise, a known negative regulator of SLC3A2 expression, *MARCH1*^37^, lost its tumor suppressor phenotype when glutamine was limiting. In contrast, the glutamine transporter *SLC1A5* (ASCT2)^38^ was substantially more essential under glutamine restriction, presumably to compensate for reduced glutamine availability. Interestingly, both *PIK3CA* and *RHEB* became more essential when glutamine was reduced, suggesting that mTORC1 relied more heavily on this arm of the pathway in the absence of SLC3A2 transporter activity. Lastly, *LZTR1*, characterized as a negative regulator of RAS^39^, no longer acted as a tumor suppressor under low glutamine conditions, and correspondingly oncogenic NRAS became less essential (Fig. 5d, e). These data suggested that RAS required SLC3A2 transporter activity to activate mTORC1, but it remained possible that glutamine restriction over two weeks was placing the cells under general metabolic stress^40^. Therefore, we tested whether acute glutamine restriction changed RAS associations with MTOR by PLA. We found that 12 hours of glutamine restriction substantially reduced RAS-MTOR PLA (Fig. 5f), defining a role for the amino acid transporter function of SLC3A2 in regulating RAS-dependent mTORC1 activation.

We next tested how amino acids regulated RAS-dependent mTORC1 signaling. RPMI 8226 and SKMM1 MM cells were transduced with control shRNA or shRNAs targeting either KRAS, NRAS or SLC3A2. Following knockdown, cells were starved of amino acids for 3 h, at which point leucine and glutamine were added back into culture or not for 90 min prior to lysis to reconstitute SLC3A2 transporter activity. Western blot analysis of mTORC1 signaling outputs found that control cells had low levels of both p70S6K (T389) and 4EBP1 (S65) phosphorylation, which was markedly enhanced by provision of leucine and glutamine (Fig. S6b). In contrast, knockdown of either RAS or SLC3A2 effectively ablated phosphorylation of p70S6K (T389) and 4EBP1 (S65) in both resting and stimulated conditions. These data demonstrate that oncogenic RAS and SLC3A2 are required for amino acid-dependent mTORC1 signaling in MM, consistent with a model where RAS commandeers mTORC1 signaling by orchestrating components of the amino acid sensing machinery.

### RAS and mTORC1 signaling in MM patients

**Fig. 6:**
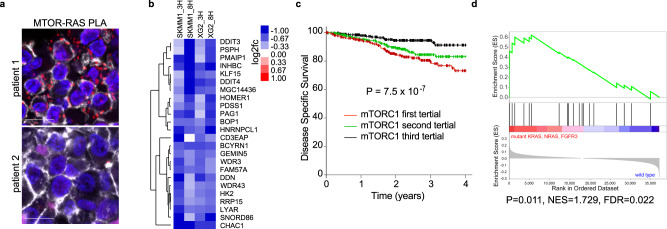
RAS-dependent mTORC1 activity in primary multiple myeloma. **a** Proximity ligation assay (PLA) between MTOR and RAS in formalin-fixed paraffin-embedded (FFPE) bone marrow aspirates from MM patients with PLA (red), CD138 (white) and DAPI (blue). Scale bar is 10 μm. **b** Changes in expression for the mTORC1 signature genes following treatment with 100 nM everolimus for the indicated cell lines and times. **c** Kaplan–Meier survival plots of MM patients from the MMRF CoMMpass trial divided into tertials by gene expression of the mTORC1 down signature in panel (**b**). The *P* value was determined using a two-sided likelihood-ratio test based on a Cox proportional hazard model with the mTORC1 signature treated as a continuous variable. **d** Gene Set Enrichment Analysis (GSEA) of mTORC1 signature for KRAS, NRAS and FGFR3 mutations in the MMRF CoMMpass patient cohort. Source data are provided as a Source Data file.

We next sought evidence of RAS-dependent mTORC1 activity in primary MM tumors. The MTOR-RAS PLA was adapted to detect MTOR and RAS interactions in formalin-fixed paraffin-embedded (FFPE) bone marrow biopsies from a cohort of 28 MM patients with known RAS mutation statuses. We observed numerous MTOR-RAS PLA puncta (Fig. 6a, red) throughout the cytosol in CD138 + cells, a marker of plasma cells (Fig. 6a, white), in a subset of MM patient samples tested. When patient samples were subdivided by RAS mutation, 33% of MM cases with *KRAS* or *NRAS* mutations (5/15; 3 KRAS, 2 NRAS) had strong MTOR-RAS PLA signals. We also found an instance where a MM case without a known RAS mutation had observable MTOR-RAS PLA in 1/13 samples (7.7%). It is possible that this patient tumor may have represented aberrant activation of wild type RAS through other mechanism, such as overexpression and/or mutation of FGFR3, which are present in ~5% of MM cases^41^.

To probe for MTOR signaling in primary MM tumors, we created a gene expression signature of mTORC1-dependent genes in RAS-dependent MM cells. SKMM1 and XG2 were treated with 100 nM everolimus and changes in gene expression relative to a DMSO control were determined at 3 and 8 hours by RNA-sequencing. Genes whose expression was decreased by an average of at least 0.5 log2fc in both cell lines were included in the signature (Fig. 6b). We applied this mTORC1 signature to gene expression data from 859 patient cases within the Multiple Myeloma Research Foundation (MMRF) CoMMpass study^42^ and determined that it was significantly correlated with disease-specific survival in this patient cohort using a Cox proportional hazard model (*p* = 7.5 × 10^−7^) (Fig. 6c), suggesting that mTORC1 signaling is correlated to poor prognosis in MM. We next performed gene set enrichment analysis (GSEA) to test if the mTORC1 signature was linked to mutations in KRAS, NRAS or FGFR3, which can activate RAS signaling independent of an oncogenic RAS mutation. Indeed, the mTORC1 signature was significantly enriched in MM samples harboring mutations in either KRAS, NRAS or FGFR3 (*P* = 0.011) (Fig. 6d), validating a link between oncogenic RAS signaling and mTORC1 activity in primary MM cases.

### Combined inhibition of mTORC1 and MEK1/2 is toxic to RAS-dependent MM

**Fig. 7:**
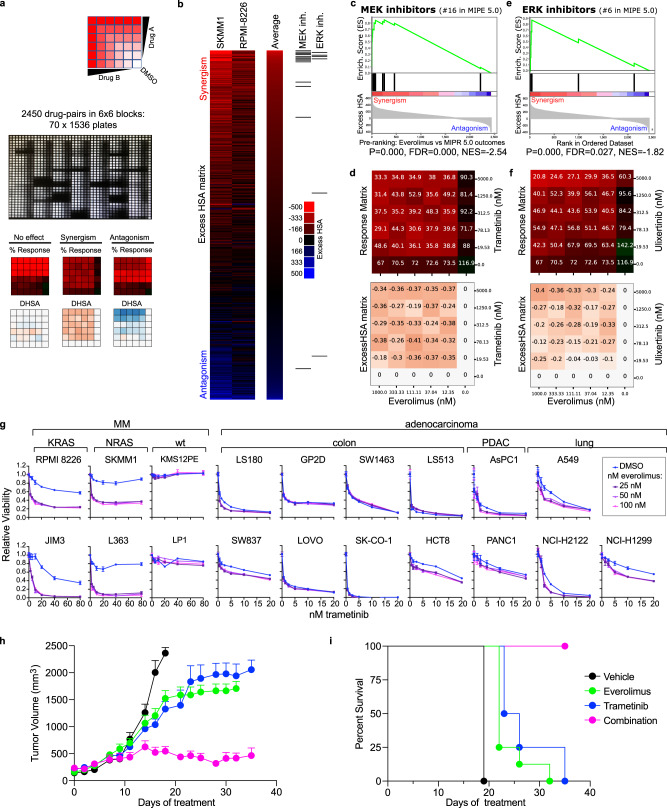
Combination therapies target RAS-dependent mTORC1 signaling. **a** Workflow for high-throughput combinatorial drug screens of everolimus treatment with the MIPE 5.0 small molecule library. **b** Heat-map view of the everolimus drug-interaction landscape in SKMM1 and RPMI 8226. Drug synergy is ranked by average aggregate excess highest single agent (HSA) values for SKMM1 and RPMI 8226. Drugs targeting MEK and ERK are indicated on the right. **c** MEK inhibitors Enrichment Plot from the Drug Set Enrichment Analysis (DSEA) of the Everolimus vs MIPE5.0 screen. The average Excess HSA (SKMM1 and RPMI 8226) was used to pre-rank combinatorial outcomes before running DSEA. *P* = 0.000; FDR = 0.000. **d** Response matrix and Excess HSA matrix for the everolimus vs. trametinib (MEKi) combination are shown (SKMM1). **e** ERK inhibitors Enrichment Plot from the DSEA of the everolimus vs. MIPE 5.0 screen. *P* = 0.000; FDR = 0.027. **f** Response matrix and Excess HSA matrix for the everolimus vs. ulixertinib (ERKi) combination are shown (SKMM1). **g** Cell viability assays for cells treated with DMSO (blue) and 25, 50 or 100 nM everolimus (purples) with the listed doses of trametinib (*x*-axis) for indicated MM or adenocarcinoma cell lines. Error bars represent SEM of the 3 technical replicates. Representative data; *n* = 3. **h** Tumor volume for SKMM1 xenografts treated with vehicle (black), 1 mg/kg trametinib (blue), 1 mg/kg everolimus (green) or the combination (pink). Representative data; *n* = 3. Error bars represent SEM of tumor size for the mice within each group. **i** Kaplan–Meier plot indicating survival for SKMM1 xenograft mice. Source data are provided as a Source Data file.

Our data indicated that RAS-dependent mTORC1 activity is a prominent feature in aggressive MM and would be an attractive therapeutic target to treat these cases, but mTORC1 inhibitors have had limited success as single agents in clinical trials^43,44^. To improve implementation of mTORC1 inhibitors in MM, we performed a high-throughput combinatorial drug screen to evaluate synergy between everolimus and the MIPE v5.0 library of 2450 mechanistically annotated, oncology-focused compounds^45^ in SKMM1 and RPMI 8226 cells in a series of 6 × 6 matrix blocks (Fig. 7a). These screens revealed exceptional synergy between everolimus and inhibitors targeting classical MAPK signaling via MEK and ERK (Fig. 7b–f; Fig. S7a, b). At the doses tested, MEK and ERK inhibitors displayed a true synthetic lethal phenotype consistent with a near de novo reliance on MEK and ERK signaling following inhibition of mTORC1 activity.

We evaluated combination therapy of everolimus with the MEK1/2 inhibitor, trametinib, in a cohort of MM lines (Fig. 7g, left). Consistent with the screen data, we found only modest growth inhibition from trametinib as a single agent (Fig. 7g, left, blue lines). However, combined treatment of trametinib and everolimus resulted in exceptional synergistic toxicity in all RAS-dependent MM lines tested (Fig. 7g, left, purple lines), yet we observed no drug synergy in RAS-independent MM lines (Fig. 7g). Unexpectedly, no drug synergy was detected in several adenocarcinoma cell lines harboring mutant KRAS (Fig. 7g, right), suggesting RAS-dependent activation of mTORC1 may be specific to MM. This drug combination resulted in apoptotic cell death in RAS-dependent MM lines, whereas everolimus or trametinib alone largely blocked the cell cycle (Fig. S8a). Consistent with this, we observed by western blot analysis that combined mTORC1 and MEK inhibition induced PARP cleavage and increased BIM expression in XG2 MM cells (Fig. S8b). We conjectured that direct activation of mTORC1 by RAS and SLC3A2 is the primary mode of oncogenic RAS signaling in MM, and that RAS may not fully engage the MAPK pathway unless mTORC1 signaling is blocked. Indeed, everolimus treatment has been previously found to increase levels of phosphorylated ERK in MM^46^.

Finally, we used mouse xenografts to determine if the combination of everolimus and trametinib retained its efficacy against MM cells in vivo. Combination therapy (pink) essentially halted tumor growth and was significantly more effective than either vehicle control (black), everolimus (green) or trametinib (blue) alone, without evidence of overt toxicity (Fig. 7h). In addition to inhibiting MM tumor growth, combination therapy extended survival compared to either vehicle or single agent-treated mice, and all combination mice were alive at the end of the treatment window (Fig. 7i). These data suggest that MM patients with tumors harboring active RAS signaling may specifically benefit from a combination of mTORC1 and MEK1/2 inhibitors.

## Discussion

**Fig. 8:**
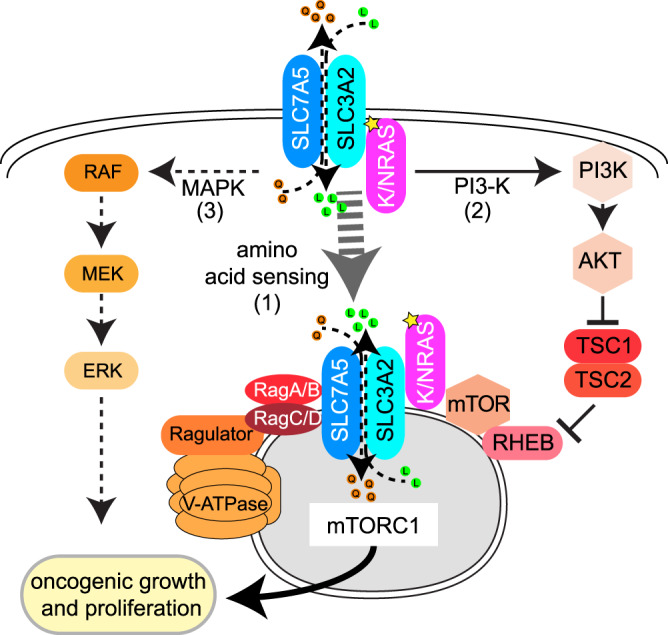
RAS co-opts mTORC1 and the amino acid sensing machinery to drive oncogenic growth in MM. Model of oncogenic RAS signaling in MM: (1) Oncogenic isoforms of RAS (KRAS or NRAS) promote localization of SLC3A2 and MTOR to endolysosomes with oncogenic RAS. (2) RAS associates with PI3-K and TSC2 to promote full activation of mTORC1 signaling. (3) RAS stimulates classical MAPK signaling.

Herein, we have described a mode of pathogenic RAS signaling in which RAS, SLC3A2 and MTOR comprise a signaling complex on endolysosomes that stimulates mTORC1 activity. These findings were unlocked by an unbiased proteogenomic approach that identified the essential interactomes of oncogenic RAS in MM, enabling the discovery of this unanticipated aspect of RAS biology. We propose a model in which RAS coordinates oncogenic growth and survival by subverting the amino acid sensing machinery through colocalization of SLC3A2 with MTOR and itself on endolysosomes to drive mTORC1 activity (Fig. 8). RAS-dependent activation of mTORC1 on endolysosomes appears to be a prevalent form of pathogenic RAS signaling in MM and is distinct from RAS obliquely activating mTORC1 through activation of PI3-K at the plasma membrane^36^. Our observations provide mechanistic insights to explain the paucity of active MEK signaling in many RAS-dependent MM tumors^7,8^ and the underwhelming clinical response to MEK inhibitors in MM patients^9,10^. However, we found that combinations of mTORC1 and MEK1/2 inhibitors were exceptionally toxic to RAS-dependent MM cell lines in vitro and nearly eliminated tumor growth in xenograft mouse models of MM. Thus, our study provides a rational basis for a combination therapy of everolimus and trametinib as an alternative to highly toxic myeloablative chemotherapy and autologous stem cell transplant in relapsed and refractory MM harboring mutant RAS.

SLC3A2 may have particular significance in MM due to the plasmacytic origin of this disease. Conditional knockout of *Slc3a2* in murine B cells resulted in a block in B cell proliferation and differentiation into plasma cells^47^. Moreover, *SLC3A2* and *SLC7A5* expression is high in plasma cells and dependent on the transcription factor *PRDM1*^48^, a master regulator of plasma cell differentiation^49^ and an essential MM gene (Fig. 1b). MM patients with high levels of SLC3A2 or SLC7A5 protein expression by immunohistological staining had inferior progression-free survival^25^, and a recent study found that SLC3A2 and SLC7A5 were among the most abundant proteins on the surface of MM cells^50^. SLC3A2 may have a prominent role in plasma cells because these cells fundamentally serve as protein production factories, secreting large quantities of antibodies that require vast reserves of amino acids^51,52^. Indeed, supplementation of glutamine to mice infected with *Plasmodium* led to increased numbers of long-lived plasma cells and more robust antibody responses against this pathogen^53^. Oncogenic RAS could have taken advantage of this distinct plasma cell biology to promote tumorigenesis in MM, and expression of mutant RAS constructs in transformed lymphoblasts has been shown to be sufficient to drive plasma cell differentiation and increase antibody secretion^54^. It remains possible that other tumor types may utilize cooperative RAS and SLC3A2. Deletion of Slc3a2 protected mice from developing tumors in a KRAS-dependent model of skin squamous cell carcinoma^55^, and knockout of *Slc7a5* prolonged survival in a *Kras*-driven mouse model of colorectal cancer^56^. However, this mode of RAS signaling may be best exemplified in MM because of its plasma cell origins.

Targeting RAS signaling has been notoriously difficult in human cancers^57^ and the recent development of drugs targeting KRAS^G12C^ mutations will not appreciably benefit MM patients since KRAS^G12C^ expression is rare in this disease^42^. Our study provides a rationale for implementing combination therapies of mTORC1 and MEK inhibitors to disrupt this unique mode of RAS signaling in MM. Similar combinations have been previously tested in clinical trials for various types of solid tumors^58,59^, but most of these trials were not successful^59^. A recent study described direct interactions between RAS and components of mTORC2 in melanoma and other solid tumor cell lines^32^. RAS-dependent mTORC2 activity would preclude the use of an mTORC1-exclusive inhibitor, and these data may explain why we observed little synergy between everolimus and trametinib in KRAS-dependent adenocarcinoma cell lines (Fig. 7g). Furthermore, these data highlight the need to consider the oncogenic cell-of-origin when designing new treatment regimens^60^. Our data suggests that malignant cells reliant on signaling through a complex composed of RAS, SLC3A2 and MTOR are acutely sensitive to mTORC1 and MEK inhibitors. Visualization of MTOR-RAS associations by PLA may serve as an excellent biomarker to identify patients with MM who would benefit from such combination therapies, even in the absence of known RAS mutations. In this regard, biochemical and mechanistic insights can drive the application of precision medicine strategies beyond simple mutational analysis.

## Methods

All research was conducted under approved biosafety protocols at the NIH (RD-20-V11-06). Human samples were studied in accordance with the ethics and principles of the Declaration of Helsinki and under Institutional Review Board approved protocols from the National Cancer Institute National Institutes of Health Protocol Review Office (11-C-0221). All samples were anonymized and de-identified. Mouse studies were performed in accordance with NCI-ACUC guidelines and under approved protocols (MB-086).

### Cell culture

MM and adenocarcinoma cell lines were grown at 37 °C with 5% CO_2_ in advanced RPMI (Invitrogen) supplemented with fetal bovine serum (Tet tested, R&D Systems), 1% pen/strep and 1% L-glutamine (Invitrogen). 293FT and 293 T cells were grown in DMEM (Invitrogen) supplemented with fetal bovine serum (Tet tested, R&D Systems), 1% pen/strep and 1% L-glutamine (Invitrogen). Cell lines were regularly tested for mycoplasma using the MycoAlert Mycoplasma Detection Kit (Lonza) and DNA fingerprinted by examining 16 regions of copy number variants^61^.

**Table:** 

Name	Description	Source	Cat. No.
ARP1C	Human multiple myeloma cell line	NCI	
EJM	Human multiple myeloma cell line	DMSZ	ACC 560
H1112	Human multiple myeloma cell line	NCI	
INA6	Human multiple myeloma cell line	DMSZ	ACC 862
JJN3	Human multiple myeloma cell line	DMSZ	ACC 541
JK6L	Human multiple myeloma cell line	DMSZ	ACC 860
KSM12PE	Human multiple myeloma cell line	JCRB	JCRB0430
KMS26	Human multiple myeloma cell line	JCRB	JCRB1187
KMS34	Human multiple myeloma cell line	JCRB	JCRB1195
L363	Human multiple myeloma cell line	DMSZ	ACC 49
LP1	Human multiple myeloma cell line	DMSZ	ACC 41
MM.1.144	Human multiple myeloma cell line	NCI	
OCI-MY5	Human multiple myeloma cell line	NCI	
RPMI 8226	Human multiple myeloma cell line	ATCC	CCL-155
SKMM1	Human multiple myeloma cell line	NCI	
XG2	Human multiple myeloma cell line	NCI	
XG6	Human multiple myeloma cell line	NCI	
NCI-H1299	Human lung adenocarcinoma cell line	ATCC	CRL-5803
NCI-H2122	Human lung adenocarcinoma cell line	ATCC	CRL-5985
A549	Human lung adenocarcinoma cell line	ATCC	CCL-185
LS180	Human colon adenocarcinoma cell line	ATCC	CL-187
SW837	Human colon adenocarcinoma cell line	ATCC	CCL-235
GP2D	Human colon adenocarcinoma cell line	Sigma	95090714
LOVO	Human colon adenocarcinoma cell line	ATCC	CCL-229
SW1463	Human colon adenocarcinoma cell line	ATCC	CCL-234
SK-CO-1	Human colon adenocarcinoma cell line	ATCC	HTB-39
LS513	Human colon adenocarcinoma cell line	ATCC	CRL-2134
HCT8	Human colon adenocarcinoma cell line	ATCC	CCL-224
ASPC1	Human pancreatic adenocarcinoma cell line	ATCC	CRL-1682
PANC1	Human pancreatic adenocarcinoma cell line	ATCC	CRL-1469
293 T	Transformed Human kidney cell line	ATCC	CRL-3216
293FT	Transformed Human kidney cell line	Thermo Fisher	R70007

### Antibodies

The following antibodies were used in this study:

**Table:** 

Antibody	Species	Clone	Supplier	Catalog No.	Lot No.	PLA	WB	Confocal	FACS
anti-pan RAS	mouse	C-4	SCBT	sc-166691	J0120	1:100	1:2000		
anti-mTOR	mouse	6H9B10	Biolegend	659202	B241067	1:50			
anti-LAMP1	mouse	H4A3	SCBT	sc20011	D1612	1:50		1:100	
anti-CD98 (SLC3A2)	rabbit		BETHYL	A304-331A		1:500			
anti-mTOR	rabbit	7C10	CST	2983	16, 19	1:200			
anti-p-4E-BP1 (S65)	rabbit		CST	4165	16		1:2000		
anti-p-p70S6K (T389)	rabbit	108D2	CST	9234	12		1:1000		
anti-4E-BP1	rabbit	53H11	CST	9644	12		1:2000		
anti-p70S6K	rabbit		CST	9202	20		1:2000		
anti-MEK1/2	rabbit	D1A5	CST	8727	5		1:2000		
anti-p-MEK1/2 (S217/221)	rabbit	41G9	CST	9154	18		1:2000		
anti-p-PKCα/β (T638/641)	rabbit		CST	9375	4		1:2000		
anti-PKCa	rabbit	D7E6E	CST	59754	1		1:2000		
anti-CD98 (SLC3A2)	rabbit	D3F9D	CST	47213	1		1:5000		
anti-GAPDH	mouse	O411	SCBT	sc-47724	E2219		1:10,000		
anti-β-actin	goat	C-11	SCBT	sc-1615	E2314		1:10,000		
anti-CD138 Alexa 647	mouse	MI15	Biolegend	356524	B271942			1:40	
anti-active Caspase 3 APC	rabbit		BD	51-68655X	8024887				1:100
anti-CD54 APC	mouse	HCD54	Biolegend	322712	B263252				1:1000
anti-CD98 (SLC3A2)	mouse	4F2	SCBT	sc-59145	E2314			1:200	1:500
anti-BioID2	mouse		Novus	nbp2-59941	CRT/17/86		1:2000	1:1000	
anti-mouse Alexa 647	goat		CST	4410	10			1:1000	
anti-rabbit Alexa 488	goat		CST	4412	18			1:1000	
anti-KRAS	mouse	F234	SCBT	sc-30	B0422	1:100	1:250	1:100	
anti-NRAS	mouse	F155	SCBT	sc-31	B1517		1:500		
anti-p-RPS6 (S240/244) Pac. Blue	rabbit	D68F8	CST	5018 S	1				1:500
anti-RPS6 Alexa 488	rabbit	54D2	CST	5317 S	9				1:500
anti-BIM	rabbit	C34C5	CST	2933 S	13		1:2000		
anti-PARP	rabbit		CST	9542	3		1:4000		

### Generation of Cas9 MM clones

MM cells were retrovirally transduced with pRetroCMV/TO-Cas9-Hygro^13^, using 293 T cells (ATCC) with helper plasmids pHIT60 and pHIT/EA6x3* in a 2:1:1 ratio in Optimem (Gibco) and Trans-IT 293 (Mirus) as previously described^13^. Cells were then selected with hygromycin and dilution cloned. Single cell clones were tested for Cas9 activity following transduction with sgRNAs for CD54 (ICAM1) or CD98 (SLC3A2) and induction with doxycycline for 10 days, at which point cells were stained with either anti-CD54 (Biolegend, clone HDCD54) or anti-CD98 (Santa Cruz Biotechnologies, clone E-5) and levels of surface expression were determined by FACS analysis.

### CRISPR essentiality screens

CRISPR screens were performed as previously described^13^. Lentivirus was produced from the Brunello sgRNA library^12^ (Addgene 73178) in 293FT cells (Invitrogen) with helper plasmids pPAX2 (Addgene 12260) and pMD2.g (Addgene 12259) in a 4:3:1 ratio in Opti-Mem (Gibco) with Trans-IT 293 T (Mirus) following the manufacturer’s instructions. 293FT supernatants were harvested at 24, 48 and 72 h, pre-cleared by centrifugation at 1000 g for 5 min and concentrated by 40X using Lenti-X concentrator (Takara) following the manufacturer’s instructions. Concentrated Brunello lentiviral library was added to Cas9 MM clones to yield ~30% infection efficiency and maintain ~1 sgRNA per cell with an average of 500 copies per sgRNA in total. Infected MM cells were selected with puromycin 3 days after viral transduction and allowed to grow under selection for another 3 days. At this point, 50 × 10^6^ cells were harvested for the day 0 timepoint and 100 ng/ml of doxycycline and 0.5 μg/ml puromycin was added to at least 50 × 10^6^ cells to induce Cas9 expression, after which a minimum of 50 × 10^6^ cells were passed every other day for 21 days to maintain an average of 500X coverage/sgRNA in the Brunello library. 50 × 10^6^ cells were harvested for the day 21 timepoint. DNA was extracted from Day 0 and 21 cell pellets with QIAmp DNA Blood Midi and Maxi kits (Qiagen).

### CRISPR sorting screens

RPMI 8226 and SKMM1 cells were transduced in duplicate with the Brunello sgRNA library as described above. Cells were selected with puromycin, and Cas9 expression was induced with doxycycline and expanded for 10 days. At this point, 50 × 10^6^ cells were collected and reserved as an input sample. The remaining ~80 × 10^6^ cells were fixed in 4% PFA for 15 min. and permeabilized with ice cold MeOH for 30 min., after which cells were washed 2X in PBS to remove MeOH and blocked in PBS with 5% FBS for 1 h. Cells were next stained with 20 ul of anti-pRPS6-Pacific Blue and 20 ul of anti-RPS6 Alexa 488 in 10 ml of PBS with 0.5% FBS for 30 min rotating at 4 °C. Cells were next washed in PBS with 0.5% FBS and resuspended at 25 × 10^6^ cells/ml in PBS with 0.5% FBS. Cells were sorted for highest and lowest 10% of p-RPS6 staining on a Sony MA900 to obtain ~2 × 10^6^ sorted cells. Cells that did not stain for total RPS6 were excluded. DNA from sorted samples was extracted using the QIAamp DNA FFPE Advanced Kit (QIAGEN) following the manufacturer’s instructions.

### CRISPR glutamine modifier screens

SKMM1 cells were transduced in duplicate with the Brunello sgRNA library as described above. Cells were selected with puromycin, and Cas9 expression was induced with doxycycline and allowed to grow for 1 week. At this point, the culture was split into 2 flasks with 50 × 10^6^ cells each. One flask was grown under normal conditions of 0.3 g/L glutamine in advanced RPMI and the other flask was grown under glutamine restriction with 10% of normal glutamine provided (0.03 g/L). Cells were grown under these conditions for an additional 2 weeks, at which point 50 × 10^6^ cells were harvested from each condition and DNA was extracted sequenced as described above.

### CRISPR Library amplification

All screens were amplified using a nested PCR strategy to first isolate sgRNA sequences from genomic DNA followed by the addition of a nextgen sequencing adapter compatible with the Illumina NextSeq2000. All products were amplified for 18 cycles per each round using ExTaq polymerase (Takara). Resultant products were size selected using a e-Gel sizeSelect 2% agarose gel (Invitrogen) and libraries were quantitated by Qubit dsDNA high-sensitivity assay (Thermo). The resulting libraries were sequenced with a NextSeq2000 (Illumina) running NextSeq 1000/2000 Control Software (v. 1.2.036376) (Illumina) and demultiplexed using DRAGEN (v.3.7.4) (Illumina) and aligned using Bowtie2 (version 2.2.9). Detailed methods and PCR primer sequences can be found here:^62^.

### CRISPR analysis

The DESeq2 algorithm^63^ was used to estimate the log-fold change of the read count between Day 21 and Day 0 samples, or treatment and control samples, of the sgRNA guides in each cell line. Of the 77,441 guides targeting genes, 9,919 (13%) were removed for having poor performance across a large number of essential gene experiments^13,64^. For each gene, the log-ratios of the remaining guides associated with that gene were averaged to estimate a gene-level, log-fold change. For each cell, these gene-level, log-fold changes were normalized by subtracting the mode of their distribution (estimated with the R-function “density”) and then divided by the root-mean- square deviation (RMSD) from that mode. Further analysis was performed using Microsoft Excel v16.63.1.

### Protein interactomes

BioID2 (^23^; Addgene 80899) with an 8X linker of GSGGG and a SnaBI site was amplified by PCR with the following primers:

BioID2 Fwd:

AATTCGAATTCCTGAAGGGCCACCatgtatccctatgatgtgccagactatgctTTCAAGAACCTGATCTGGCTGAAGG

BioID2 Rev:

cgccggccctcgaggtacgtactaAGCGCTTCTTCTCAGGCTGAAC

The PCR fragment was purified and cloned into the StuI site in the MCS of pBMN-LYT2. The resulting BioID2-8Xlinker-pBMN-LYT2 vector permitted the addition of a BioID2-linker to the amino terminus of any gene of interest by inserting a gene at the SnaBI site using Gibson cloning (New England Biolabs). Synthetic gene fragments (G-block, IDT) of KRAS^G12V^ and NRAS^G12V^ or PCR amplicons of SLC3A2 derived from cDNA from germinal center B cells^13^ were cloned into this vector:

KRAS^G12V^

CTGCCGGATCCGAATTCTAGCCACAatgactgaatataaacttgtggtagttggagctgTtggcgtaggcaagagtgccttgacgatacagctaattcagaatcattttgtggacgaatatgatccaacaatagaggattcctacaggaagcaagtagtaattgatggagaaacctgtctcttggatattctcgacacagcaggtcaagaggagtacagtgcaatgagggaccagtacatgaggactggggagggctttctttgtgtatttgccataaataatactaaatcatttgaagatattcaccattatagagaacaaattaaaagagttaaggactctgaagatgtacctatggtcctagtaggaaataaatgtgatttgccttctagaacagtagacacaaaacaggctcaggacttagcaagaagttatggaattccttttattgaaacatcagcaaagacaagacagagagtggaggatgctttttatacattggtgagggagatccgacaatacagattgaaaaaaatcagcaaagaagaaaagactcctggctgtgtgaaaattaaaaaatgcattataatgGTAGGTGGAGGCGGGTCGGG

NRAS^G12V^

CTGCCGGATCCGAATTCTAGCCACAatgactgagtacaaactggtggtggttggagcagttggtgttgggaaaagcgcactgacaatccagctaatccagaaccactttgtagatgaatatgatcccaccatagaggattcttacagaaaacaagtggttatagatggtgaaacctgtttgttggacatactggatacagctggacaagaagagtacagtgccatgagagaccaatacatgaggacaggcgaaggcttcctctgtgtatttgccatcaataatagcaagtcatttgcggatattaacctctacagggagcagattaagcgagtaaaagactcggatgatgtacctatggtgctagtgggaaacaagtgtgatttgccaacaaggacagttgatacaaaacaagcccacgaactggccaagagttacgggattccattcattgaaacctcagccaagaccagacagggtgttgaagatgctttttacacactggtaagagaaatacgccagtaccgaatgaaaaaactcaacagcagtgatgatgggactcagggttgtatgggattgccatgtgtggtgatgGTAGGTGGAGGCGGGTCGGG

SLC3A2 Fwd:

CTGCCGGATCCGAATTCTAGCCACAatggagctacagcctcctgaag

SLC3A2 Rev:

CCCGACCCGCCTCCACCTACtcaggccgcgtaggggaagcg

Resultant BioID2 constructs were packaged into retrovirus using 293 T cells (ATCC) with helper plasmids pHIT60 and pHIT/EA6x3* in a 2:1:1 ratio in Optimem (Gibco) with Trans-IT 293 (Mirus) as previously described^13^. Transduced MM cells were purified with anti-Lyt2 (mouse CD8) magnetic beads (Dynal/Thermo), and purified cells were grown in SILAC media, containing amino acids labeled with stable isotopes of arginine and lysine, for 2 weeks and then expanded to 50 × 10^6^ cells. In certain cases, cells were infected with pLKO-shKRAS.2 or pLKO-shNRAS.1 (see below). 16 h prior to lysis, biotin (Sigma) was added to a final concentration of 50 μM to transduced cells. Cells were then lysed at 1 × 10^7^ cells per ml in RIPA buffer modified for MS analysis (1% NP-40, 0.5% deoxycholate, 50 mM Tris, pH 7.5, 150 mM NaCl, 1 mM Na_3_VO_4_, 5 mM NaF, 1 mM AEBSF) for 10 min. on ice. Lysates were cleared by centrifugation at 14,000xg for 20 min. at 4 °C. 35μl of pre-washed streptavidin agarose beads (Thermo) were added to each sample; samples were then rotated at 4 °C for 2 hours, then washed three times in 1X RIPA buffer, then solubilized with 4X LDS sample buffer (Invitrogen) with 1% Nupage reducing agent (Invitrogen), and boiled for 5 min.

For MS analysis, proteins were separated by one-dimensional gel electrophoresis (4–12% NuPAGE Bis-Tris Gel; Invitrogen), and the entire lane of a Coomassie blue-stained gel was cut into 23 slices. All slices were processed as described previously^65^. After tryptic digestion of the proteins the resulting peptides were resuspended in sample loading buffer (2% acetonitrile and 0.05% trifluoroacetic acid) and were separated by an UltiMate 3000 RSLCnano HPLC system (Thermo Fisher Scientific) coupled online to a Q Exactive HF mass spectrometer (Thermo Fisher Scientific). First, peptides were desalted on a reverse phase C18 precolumn (Dionex 5 mm length, 0.3 mm inner diameter) for 3 minutes. After 3 minutes the precolumn was switched online to the analytical column (30 cm length, 75 mm inner diameter) prepared in-house using ReproSil-Pur C18 AQ 1.9 mm reversed phase resin (Dr. Maisch GmbH). Buffer A consisted of 0.1 % formic acid in H_2_O, and buffer B consisted of 80% acetonitrile and 0.1% formic acid in H_2_O. The peptides eluted from buffer B (5–42% gradient) at a flow rate of 300 nl/min over 76 min. The temperature of the precolumn and the analytical column was set to 50 °C during the chromatography. The mass spectrometer was operated in a TopN data-dependent mode, where the 30 most intense precursors from survey MS1 scans were selected with an isolation window of 1.6 Th for MS2 fragmentation under a normalized collision energy of 28. Only precursor ions with a charge state between 2 and 5 were selected. MS1 scans were acquired with a mass range from 350 to 1600 m/z at a resolution of 60,000 at 200 m/z. MS2 scans were acquired with a starting mass of 110 Th at a resolution of 15,000 at 200 m/z with maximum IT of 54 ms. AGC targets for MS1 and MS2 scans were set to 1E6 and 1E5, respectively. Dynamic exclusion was set to 20 s.

### MS data analysis

MS data analysis was performed using the software MaxQuant (version 1.6.0.1) linked to the UniProtKB/Swiss-Prot human database containing 155,990 protein entries and supplemented with 245 frequently observed contaminants via the Andromeda search engine^66^. Precursor and fragment ion mass tolerances were set to 6 and 20 ppm after initial recalibration, respectively. Protein biotinylation, N-terminal acetylation and methionine oxidation were allowed as variable modifications. Cysteine carbamidomethylation was defined as a fixed modification. Minimal peptide length was set to 7 amino acids, with a maximum of two missed cleavages. The false discovery rate (FDR) was set to 1% on both the peptide and the protein level using a forward-and-reverse concatenated decoy database approach. For SILAC quantification, multiplicity was set to two or three for double (Lys+0/Arg+0, Lys+8/Arg+10) or triple (Lys+0/Arg+0, Lys+4/Arg+6, Lys+8/Arg+10) labeling, respectively. At least two ratio counts were required for peptide quantification. The “re-quantify” option of MaxQuant was enabled. Data was filtered for low confidence peptides.

### Phosphoproteome Analysis

SKMM1 cells were grown and expanded in SILAC media to 100 × 10^6^ cells per condition. At this point, cells were transduced with concentrated lentivirus: cells in ‘Light’ media were transduced with shCtrl, ‘Medium’ were transduced with shNRAS.1 and ‘Heavy’ were transduced with shNRAS.2 (See below). Cells were selected with puromycin (Invitrogen) the following day and allowed to grow under selection conditions for 2 days, after which they were lysed in 1% NP-40, 50 mM Tris, pH 7.5, 150 mM NaCl, 1 mM Na_3_VO_4_, 5 mM NaF with 1 tablet/10 ml EDTA-free protease inhibitor cocktail tablets (Roche). Changes in global phosphorylation were analyzed as previously described^67^. For analysis, the log2-fold change of shNRAS.1 and shNRAS.2 were averaged.

### Pathway analysis

Pathway analysis was performed using ToppFun from the ToppGene Suite (https://toppgene.cchmc.org)^68^. Gene Ontogeny Biological Process and Pathway analysis were used with the indicated gene lists and log2fc values.

### shRNA and sgRNA mediated knockdown

Individual shRNAs were obtained from the MISSION shRNA Library from the RNAi Consortium TRC1.0 in the pLKO.1 vector (SIGMA):

shCTRLGCCAAGATTCAGAATCCCAAA

shNRAS.1GAAACCTGTTTGTTGGACATA

shNRAS.2CAGTGCCATGAGAGACCAATA

shKRAS.1GCAGACGTATATTGTATCATT

shKRAS.2GAGGGCTTTCTTTGTGTATTT

shSLC3A2.1CGAGAAGAATGGTCTGGTGAA

shSLC3A2.2GCTGGGTCCAATTCACAAGAA

shRPTOR.1CCTCACTTTATTTCCATGTAA

shRPTOR.2CGAGTCCTCTTTCACTACAAT

shRICTOR.1GCACCCTCTATTGCTACAATT

shRICTOR.2CGTCGGAGTAACCAAAGATTA

sgCTRLTTGCAATGCTGCTATAGAAG

sgTSC2CAGCATCTCATACACACGCG

Lentiviral transductions of shRNAs were performed as described above. Transduced MM cells were selected with 1μg/ml puromycin (Gibco) for 2 days before either western blot analysis or proximity ligation.

### mNeonGreen fusions

For co-immunoprecipitation and imaging studies, mutant RAS isoforms of KRAS^G12D^, KRAS^S17N^, NRAS^G12D^, NRAS^Q61L^ and NRAS^S17N^ were linked to mNeonGreen as gene fragments (Twist Biosciences). Sequences of these gene fragments can be found in the accompanying Source Data file. Gene fragments were cloned into pBMN-LYT2 at the StuI sites. MM cells were retrovirally transduced and selected for LYT2 expression, as described above. For lysis, 20 × 10^6^ cells were lysed in 0.5% CHAPS (for co-IP of SLC3A2) or 0.3% CHAPS (for co-IP of MTOR, RPTOR and RICTOR) in lysis buffer (50 mM Tris, pH 7.5, 150 mM NaCl, 1 mM Na_3_VO_4_, 5 mM NaF, 1 mM AEBSF) for 10 min. on ice, and lysates were cleared by centrifugation at 14,000xg for 20 min. at 4 °C and the post-nuclear supernatant was collected. Samples were divided in two and 25 μl of mNeonGreen-Trap agarose (Chromotek) was added to pulldown mNeonGreen-tagged RAS constructs, or 25 μl of saturated control beads (Chromotek), after which lysates were rotated at 4 C for 2 hours. Beads were then washed 3X in CHAPS lysis buffer and 30 μl 2X Laemmli sample buffer (BioRad) was added to each sample, followed by boiling for 5 min. Samples were then subjected to western blot analysis as described below.

### Proximity Ligation Assay

MM cells shRNAs plated onto a 15 well μ-Slide Angiogenesis ibiTreat chamber slide (Ibidi) and allowed to adhere to the surface for 1 hour at 37 °C. In certain cases, cells were transduced with shRNAs targeting genes (described above) or were overexpressing mNeonGreen-KRAS and mNeonGreen-NRAS mutants (described above). Cells were next fixed with 4% paraformaldehyde (Electron Microscopy Sciences) for 20 min at room temperature and then washed in PBS (Invitrogen). Cellular membranes were labeled with 5 μg/ml wheat germ agglutinin (WGA) conjugated to Alexa Fluor 488 (Thermo Fisher Scientific) for 10 min at room temperature. Cells were permeabilized in cold methanol for 20 min, washed in PBS and then blocked in Duolink Blocking buffer (Sigma) for 1 hour at room temperature. Primary antibodies were diluted in Duolink Antibody Diluent (Sigma) and incubated overnight at 4 °C (See above). Cells were next washed for 20 min in TBST with 0.5% tween-20, followed by addition of the appropriate Duolink secondary antibodies (Sigma), diluted and mixed according to the manufacturer’s instructions. Cells were incubated for 1 h at 37 °C, after which cells were washed in TBST with 0.5% tween-20 for 10 min. For studies examining immunofluorescence and PLA simultaneously, we incubated samples with labeled anti-mouse and anti-rabbit secondary antibodies for 1 hour at room temperature, followed by 10 min. wash in TBST with 0.5% tween-20. Ligation and amplification steps of the PLA were performed using the Duolink in situ Detection Reagents Orange kit (Sigma) according to the manufacturer’s instructions. Following the PLA, cells were mounted in Fluoroshield Mounting Medium with DAPI (Abcam). Images were acquired on a Zeiss LSM 880 Confocal microscope using Zeiss Zen Black version 2.3. Images for display were prepared with NIH ImageJ/FIJI software version 2.0.0-rc-65/1.5ls^69^. PLA spots were counted using Blobfinder version 3.2^70^. Cells with small nuclei that were possibly apoptotic (area below 750 pixels) were excluded from analysis. PLA Score was determined by normalizing the number of PLA spots counted in each sample to the average number of PLA spots counted in the control sample, which was set to 100. Box and whisker plots display the median PLA Score with whiskers incorporating 10–90% of all data; outliers are displayed as dots. Statistical comparisons were made by one-way ANOVA with Dunnett’s post test or by unpaired Mann–Whitney t test using Prism 9.

The PLA was performed on formalin-fixed, paraffin-embedded (FFPE) tissue microarrays or biopsy samples in a similar manner. Samples were deparaffinized in xylene and rehydrated in graded alcohol and distilled water. Heat induced antigen retrieval was performed at pH 6.0 for 30 minutes. Slides were then placed in tris-buffered solution and prepared for proximity ligation assay, as described above, MM samples were co-stained with mouse anti-human CD138-Alexa647 (Biolegend, clone MI15). PLA was scored manually in CD138+ cells in a blinded fashion as either – or +. All primary patient samples were anonymized or de-identified before PLA analysis.

### Human Samples

All cases were either needle aspirates from bone marrow or bone marrow aspirate clots. Samples were fixed in 10% buffered formalin for 18-24 hours and paraffin embedded for long term storage. Samples were studied in accordance with the ethics and principles of the Declaration of Helsinki and under Institutional Review Board approved protocols from the National Cancer Institute National Institutes of Health Protocol Review Office (protocol number 11-C-0221). Informed consent was obtained from all patients or samples were given an IRB-waiver as archived tissue submitted for consultation to the Department of Laboratory Medicine. All samples were anonymized or de-identified for subsequent PLA analysis. Participants were not compensated.

### Immunofluorescence

RPMI 8226 cells were transduced with pBMN-Lyt2-mNeonGreen-KRASG^12D^ or SKMM1 cells with pBMN-Lyt2-mNeonGreen-NRAS^G12D^. Transduced cells were enriched with anti-mouse CD8 (Lyt2) dynal beads (Thermo) and allowed to recover for 2 days. For live cell imaging, cells were labeled with 100 nM Lysotracker Red (Thermo) for 1 hour. To label cytosolic proteins, cells were plated on ibidi u-Slide 8 well chamber slides (ibidi) for 1 hour, then fixed with 4% paraformaldehyde (Electron Microscopy Sciences) for 20 min at room temperature and then permeabilized with cold methanol for 30 min. Cells were next washed 2X with PBS and blocked with PBS + 5% BSA for 1 h. Cells were stained with indicated primary antibodies for 2 h at room temperature, and following 15 min wash in tris-buffered saline with 0.05% tween cells, were stained with matching secondary antibodies for 1 hour at room temperature, followed by 15 min wash with tris-buffered saline with 0.05% tween, and 1 min wash in PBS. Cells were then placed in mounting media without DAPI (ibidi). All samples were imaged on a Zeiss LSM 880 as described above.

### Western blot analysis

Cells were then lysed at 1 × 10^7^ cells per ml in modified RIPA buffer (1% NP-40, 0.5% deoxycholate, 50 mM Tris, pH 7.5, 150 mM NaCl, 1 mM Na_3_VO_4_, 5 mM NaF, 1 mM AEBSF) for 10 min. on ice. Lysates were cleared by centrifugation at 14,000 xg for 20 min. at 4 °C, and the post-nuclear supernatant was collected. Protein concentrations were determined using the Pierce BCA protein assay kit (Thermo) according to the manufacturers protocol. 100 ul of lysate and 40 μl of 4X Laemmli sample buffer (BioRad) with 1% β-mercaptoethanol (BioRad) were combined and then boiled for 5 min. 15 μg of each lysate was run on a 4–12% gradient gel (BioRad) and transferred to a PVDF membrane (Millipore) on an Owl semi-dry transfer device (Thermo). PVDF membranes were blocked with 5% milk (BioRad) in TBST and then probed with listed antibodies diluted in either 1% BSA (anti-phospho-specific antibodies; MPI) or milk and anti-rabbit-HRP or anti-mouse-HRP (Cell Signaling Technology) where appropriate. Blots were imaged with a ChemiDoc Imaging System (Bio-Rad) using Image Lab Touch Software (v2.3.0.07) (Bio-Rad).

### Amino acid stimulations

For amino acid stimulations, RPMI 8226 and SKMM1 cells were transduced with shRNAs as described above. Two days after puromycin selection, cells were washed twice with PBS to remove growth media and cells were re-plated with Tyrode’s buffer (120 mM NaCl, 5 mM KCl, 25 mM HEPES, 2 mM CaCL_2_, 2 mM MgCL_2_, 6 g/L glucose, pH 7.4) with 4% dialyzed FBS (Sigma). Cells were grown at 37 °C for 3 h under these conditions. At this point, 10^6^ cells were placed in 1 ml Tyrode’s:FBS, and cells were either left unstimulated or provided 10 μl of 100X leucine/glutamine (final concentration: L-leucine 50 mg/L (Sigma); L-glutamine 300 mg/L (Sigma)). Cells were then incubated at 37 °C for 90 minutes, at which point they were lysed in SDS sample buffer and subjected to western blot analysis as described above. For drug experiments, all drugs were obtained from Selleckchem and used at the indicated concentrations.

### FACS analysis

MM cell lines were transduced with sgCD54 vector co-expressing GFP and stained with anti-CD54 (1:1000) to select Cas9-expressing cells as previously described^13^. SLC3A2 surface expression was measured by staining 2 × 10^5^ cells on ice with anti-CD98 (1:500) for 20 minutes in FACS buffer (PBS with 2% BSA). Cells were washed with FACS buffer and stained with anti-mouse-Alexa647 (1:1000; CST) for 20 minutes on ice, then washed again and resuspended in 250 μl of FACS buffer. These cells were analyzed on a BD FACS Calibur using CellQuest Pro version 6.0 and analyzed with FlowJo version 10. For cell cycle analysis, cells were treated for 1 day with DMSO, 50 nM everolimus (Selleckchem), 5 nM trametinib (Selleckchem) or both drugs together. Treated cells were stained with propidium iodide (Invitrogen) following the manufacturer’s protocol. For cell viability analysis, cells were treated for 2 days under the same conditions and then stained with either 7AAD (Invitrogen) and Annexin V-PE (BD) or stained with anti-cleaved caspase 3 Alexa 647 (BD), following the manufacturer’s protocol. Stained cells were analyzed with a CytoFLEX LX (Beckman Coulter) and data was analyzed with FlowJo version 10.

### Drug Sensitivity Assays

MM cell lines were seeded at ~5000 cells/well in triplicate in 96-well plates. Trametinib and everolimus (SelleckChem) dissolved in DMSO were diluted in equal volumes at the indicated concentrations. Cells were cultured with drugs for 4 days. Drugs were replenished after 48 h. Metabolic activity was measured at day 4 with CellTiter 96 (Promega) following the manufacturer’s protocol. Absorbance was measured at 490 nm using a 96-well Tecan Infinite 200 Pro plate reader.

### Gene expression profiling and signature enrichment

SKMM1 and XG2 MM cells were treated with 100 nM everolimus and harvested at indicated times after shRNA induction. RNA was extracted using the AllPrep kit (QIAGEN) and RNA libraries were prepared using the TruSeq V3 chemistry (Illumina) according to the manufacturer’s protocol. Sequencing of libraries was done on a NovaSeq S1 with a read length of 2 × 100 bp. Alignment to the human genome (hg19) was done using STAR-aligner. Normalized reads were Log2 transformed to calculate Digital Gene Expression values, as previously described^64^. Changes in gene expression between everolimus treated and DMSO control cells were determined, and genes with an average log2 fold change of less than −0.5 per in both cell lines were included in the mTORC1 signature. *P* values for differences in signature averages were calculated using a two-sided *t*-test. *P* values for the association between mTORC1 and survival were from a two-sided likelihood-ratio test based on a Cox proportional hazard model with the mTORC1 signature treated as a continuous variable.

### CoMMpass data

Data from the MMRF CoMMpass dataset^42^ was downloaded through GDC portal using GDC-client tool and processed the GDC standard pipelines (https://docs.gdc.cancer.gov/Data/Bioinformatics_Pipelines/DNA_Seq_Variant_Calling_Pipeline/). Then, processed WES result was further annotated as previously described^64^ to call gene mutations. RNA-Seq was further processed and normalized as previously described^64^.

### GSEA

Gene Set Enrichment Analysis (GSEA)^71^ was performed on all COMMPASS RNA-seq samples comparing the K-RAS/N-RAS/FGFR3 mutant samples to all others. Significant enrichment was observed for the functionally defined gene set of mTORC1-responsive genes.

### Quantitative high-throughput combination screening (qHTCS)

Drug combination screening was performed as previously described^72^. Briefly, 10 nL of compounds were acoustically dispensed into 1536-well white polystyrene tissue culture-treated plates with an Echo 550 acoustic liquid handler (Labcyte). Cells were then added to compound-containing plates at a density of 500-cells/well in 5 μL of medium. A 5-point custom concentration range, with constant 1:4 dilution was used for all the MIPE 5.0 drugs^45^ in the primary 6 × 6 matrix screening against Everolimus (1:3 dilution), and a 9-point custom concentration range was used for secondary validation in 10 × 10 matrix format.

Plates were incubated for 48 h at standard incubator conditions covered by a stainless steel gasketed lid to prevent evaporation. 48 h post compound addition, 3 μL of Cell Titer Glo (Promega) were added to each well, and plates were incubated at room temperature for 15 minutes with the stainless-steel lid in place. Luminescence readings were taken using a Viewlux imager (PerkinElmer) with a 2 s exposure time per plate.

### Drug-Target Set Enrichment Analysis (DTSEA)

To enable the unbiased identification of over-represented drug targets that synergized with mTOR inhibition (Everolimus) in MM cell-lines, we used the Excess over the Highest Single Agent (ExcessHSA) metric to quantitatively assess synergism and antagonism throughout the Everolimus vs MIPE 5.0 combination screenings. We then ranked the entire MIPE 5.0 drug-universe based on the average ExcessHSA score in SKMM-1 and RPMI-8226 cells.

We used this ranked list to run a pre-ranked Drug-Target Set Enrichment Analysis (DTSEA), against a custom collection of drug-target sets representing any MIPE 5.0 drug-target that is covered by at least 3 small-molecule drugs (*n* = 278). The pre-ranked enrichment analysis was performed using the GSEA software (v4.0.3)^71^ with a weighted enrichment statistic.

### Xenografts

All mouse experiments were approved by the National Cancer Institute Animal Care and Use Committee (NCI-ACUC) and were performed in accordance with NCI-ACUC guidelines and under approved protocols. Female NSG (non-obese diabetic/severe combined immunodeficient/common gamma chain deficient) mice were obtained from NCI Fredrick Biological Testing Branch and used for the xenograft experiments between 6–8 weeks of age. Mice were housed in specific pathogen-free facility in ventilated microisolator cages with 12 h light and 12 h dark cycles at 72 F and 40–60% relative humidity. Approved protocols allowed tumor growth below 20 mm in any dimension; no animals had tumors which exceeded these limits. Female NSG (non-obese diabetic/severe combined immunodeficient/common gamma chain deficient) mice were obtained from NCI Fredrick Biological Testing Branch and used for the xenograft experiments between 6–8 weeks of age. SKMM1 multiple myeloma tumors were established by subcutaneous injection of 10^6^ cells in a 1:1 Matrigel/PBS suspension. Treatments were initiated when tumor volume reached a mean of 200 mm^3^. MEK inhibitor (trametinib; Selleckchem) was prepared in 10% (v/v) DMSO + 90% (v/v) corn oil and administered p.o. once per day (1 mg/kg/day). mTORC1 inhibitor (everolimus; Selleckchem) was prepared in 10% (v/v) DMSO + 30% (v/v) propylene glycol + 5% (v/v) Tween 80 + 55% (v/v) H_2_O and administered p.o. once per day (1 mg/kg/day). For the MEK/MTOR inhibitor combination, drugs were given at the same concentration and schedule as single agents. Each treatment group contained between 3 and 8 mice. Tumor growth was monitored every other day by measuring tumor size in two orthogonal dimensions and tumor volume was calculated by the following equation: tumor volume = (length × width^2)/2.

### Reporting summary

Further information on research design is available in the Nature Research Reporting Summary linked to this article.

## Supplementary information

Supplementary Figures

Reporting Summary

## Data Availability

Mass spectrometry data was uploaded to the Proteomics Identification Database (PRIDE) under accession number PXD031662. RNA seq data was uploaded to the Gene Expression Omnibus (GEO) under accession number GSE196231. The CRISPR screen score (CSS) data used in this study are provided in the Source Data file. Raw images available upon request. Public databases used herein can be found at https://www.uniprot.org/proteomes/UP000005640 and https://toppgene.cchmc.org. Source data are provided with this paper.

## Source data

Source Data
