# Publisher Correction: Oncogenic RAS commandeers amino acid sensing machinery to aberrantly activate mTORC1 in multiple myeloma

**DOI:** 10.1038/s41467-022-33618-w

**Published:** 2022-10-03

**Authors:** Yandan Yang, Arnold Bolomsky, Thomas Oellerich, Ping Chen, Michele Ceribelli, Björn Häupl, George W. Wright, James D. Phelan, Da Wei Huang, James W. Lord, Callie K. Van Winkle, Xin Yu, Jan Wisniewski, James Q. Wang, Frances A. Tosto, Erin Beck, Kelli Wilson, Crystal McKnight, Jameson Travers, Carleen Klumpp-Thomas, Grace A. Smith, Stefania Pittaluga, Irina Maric, Dickran Kazandjian, Craig J. Thomas, Ryan M. Young

**Affiliations:** 1 Lymphoid Malignancies Branch, Center for Cancer Research, National Cancer Institute, National Institutes of Health, Bethesda, MD 20892 USA; 2 Department of Medicine II, Heamatology/Oncology, Goethe University, 60323 Frankfurt, Germany; 3 Division of Preclinical Innovation, National Center for Advancing Translational Sciences, National Institutes of Health, Rockville, MD 20850 USA; 4 Biometric Research Branch, DCTD, National Cancer Institute, National Institutes of Health, Bethesda, MD 20892 USA; 5 Experimental Immunology Branch, National Cancer Institute, National Institutes of Health, Bethesda, MD 20892 USA; 6 Laboratory of Pathology, Center for Cancer Research, National Cancer Institute, National Institutes of Health, Bethesda, MD 20892 USA; 7 Hematology Service, Department of Laboratory Medicine, National Institutes of Health Clinical Center, Bethesda, MD 20892 USA; 8 Department of Medicine, University of Miami Health System, Miami, FL 33136 USA

**Keywords:** Myeloma, Cell signalling, Oncogenes

Correction to: *Nature Communications* 10.1038/s41467-022-33142-x, Article published online 17 September 2022

In this article, Yandan Yang, Arnold Bolomsky, Thomas Oellerich, and Ping Chen should have been denoted as equally contributing authors. The original article has been corrected.

