# Supplementary material for: Oncogenic RAS commandeers amino acid sensing machinery to aberrantly activate mTORC1 in multiple myeloma
Source: Nat Commun. 2022 Sep 17;13:5469. doi: 10.1038/s41467-022-33142-x (PMC9482638; doi:10.1038/s41467-022-33142-x)
Supplement: Supplementary file 1 — Supplementary Figures [file 41467_2022_33142_MOESM1_ESM.pdf]

Supplementary Figures

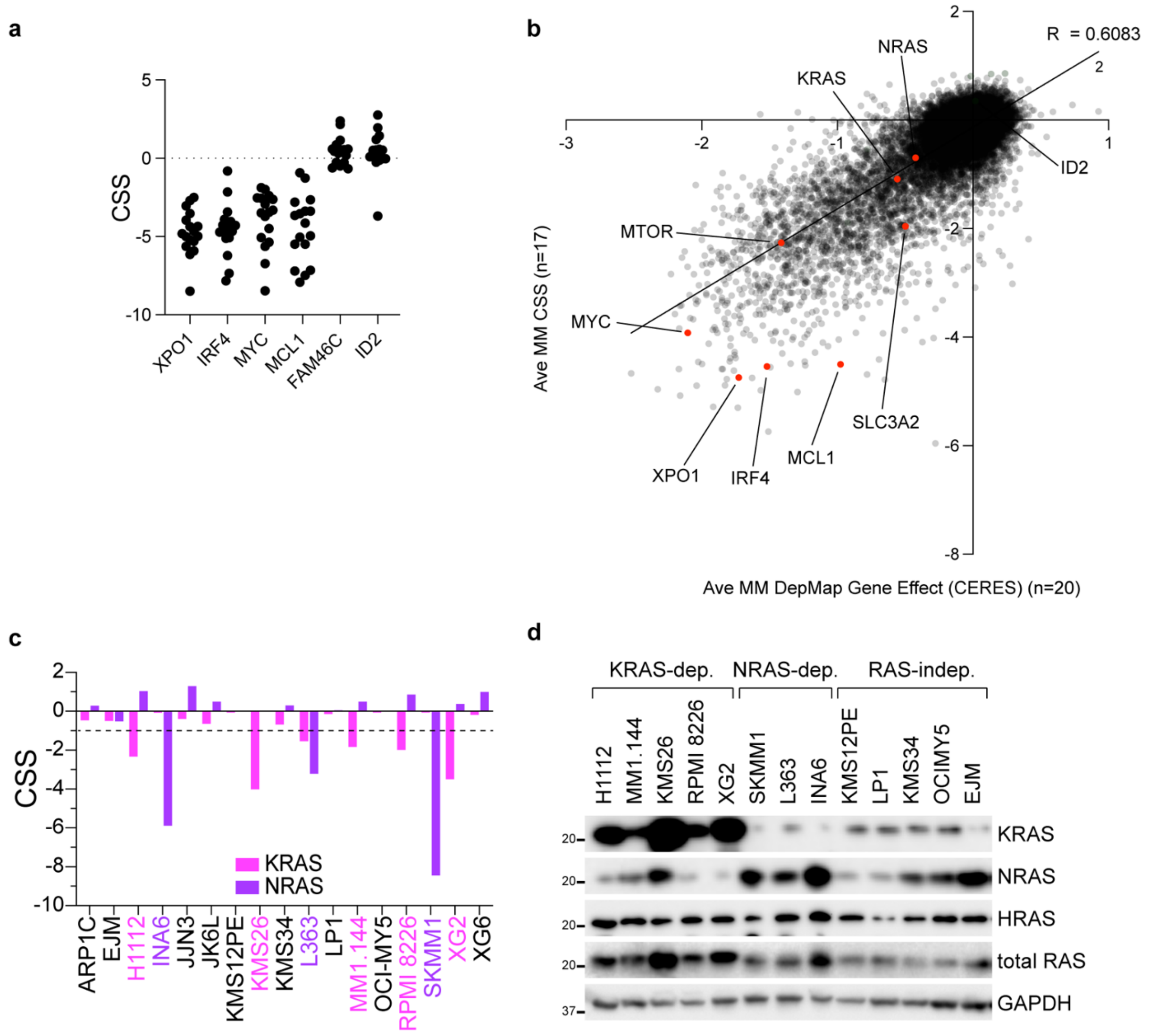

Supplemental Figure 1. CRISPR screens identify essential and tumor suppressor genes in multiple myeloma. **a** CRISPR screen score (CSS; y-axis) for indicated genes. Each dot represents an individual MM cell line from the cohort of 17 cell lines identified in Fig. 1A. **b** Correlation between CRISPR screens. Average CERES score for the DepMap cohort of 20 MM cell lines (x-axis) plotted by the average CSS for the 17 MM cell lines in this study. Genes from Fig. S1A, as well as NRAS, KRAS and SLC3A2, highlighted in red. **c** Identification of KRAS and NRAS dependent MM lines by CSS (y-axis). **d** Western blot of RAS isoforms in KRAS-dependent, NRAS-dependent and RAS-independent cell lines. Representative blots; n=2. Source data are provided as a Source Data file.

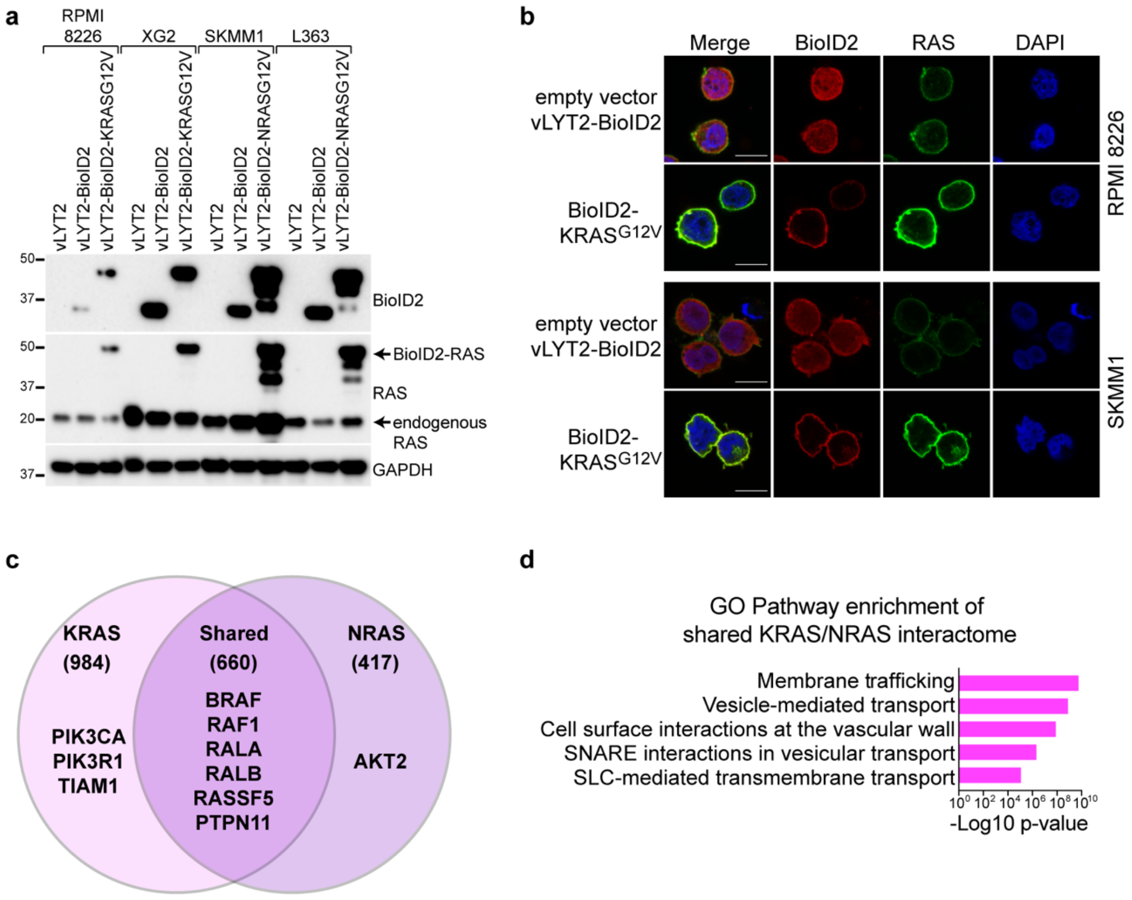

Supplemental Figure 2. Proteomic determination of mutant KRAS and NRAS protein interactomes. **a** Western blot analysis of BioID2 constructs in MM cell lines, n=1. **b** Immunofluorescence of BioID2 constructs in RPMI 8226 and SKMM1 cells with anti-BioID2 (red), anti-RAS (green) and DAPI (blue). Scale bar is 10 µm; n=1. **c** Venn diagram depicting the total size of the BioID2-KRAS^{G12V} and BioID2-NRAS^{G12V} interactomes in MM cell lines (≥1.0 log2fc), as described in main text. Canonical RAS effectors are listed. **d** Gene Ontology pathway enrichment of the shared KRAS/NRAS (≥2.0 log2fc) interactome, with inverse log10 P-values with Bonferroni correction plotted on the x-axis. Source data are provided as a Source Data file.

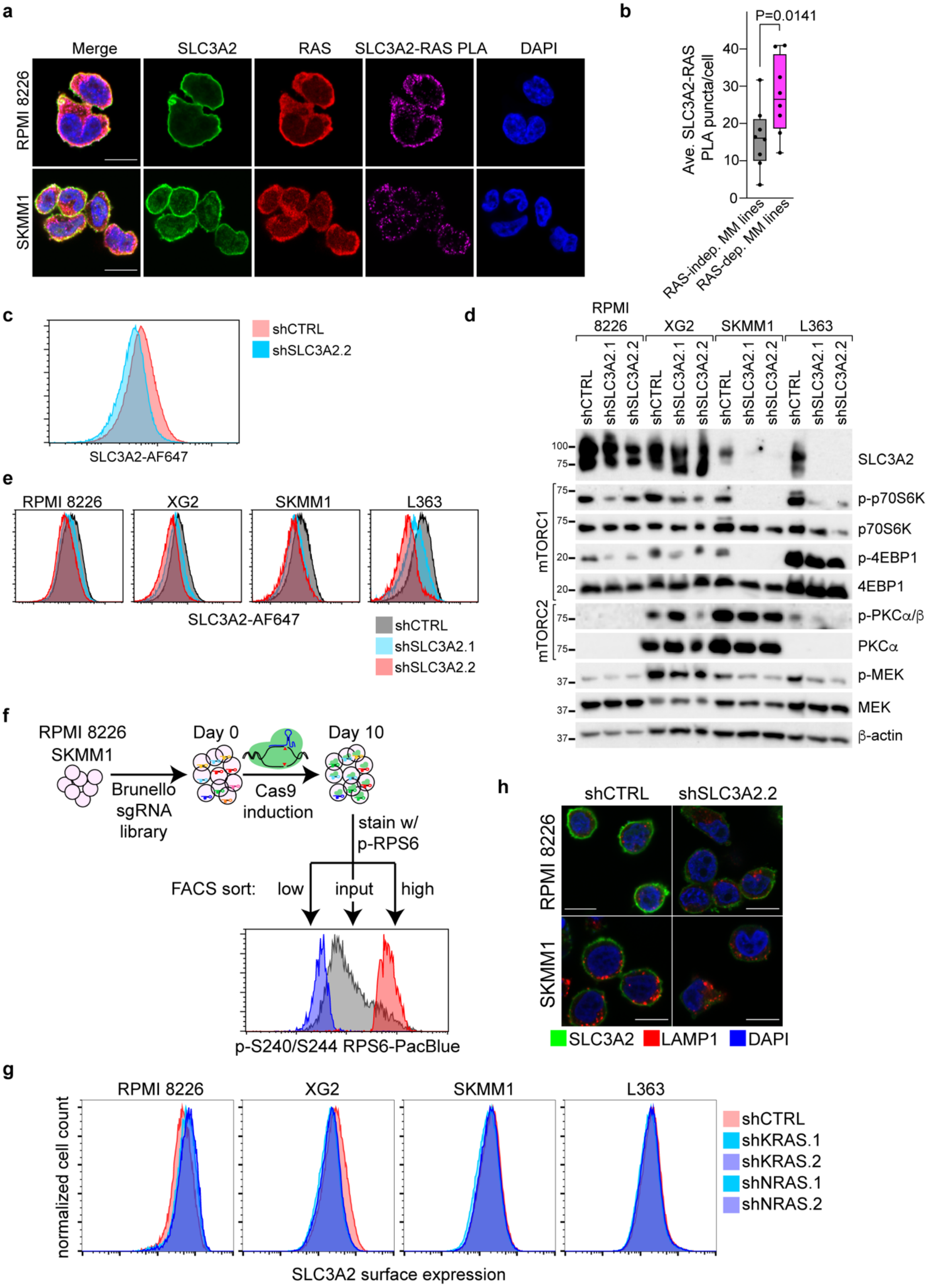

Supplemental Figure 3. SLC3A2 and RAS associate and regulate mTORC1. **a** Combined proximity ligation assay (PLA) and immunofluorescence imaging of SLC3A2 (green) and RAS (red)

in RPMI 8226 and SKMM1 cells with SLC3A2-RAS PLA (pink) and DAPI (blue) staining. Scale bar is 10 μm. Representative images; n=2. **b** The average SLC3A2-RAS PLA puncta/cell for 16 MM cell lines. Each dot represents an individual MM line. P-value from one-tailed Mann-Whitney test. Representative data, n=2. **c** FACS analysis of SLC3A2 knockdown in SKMM1 cells from Fig. 2E. **d** Western blot analysis of mTORC1 and mTORC2 effectors in MM lines transduced with shCTRL, or SLC3A2-specific shRNAs. Representative blots; n=6. **e** FACS analysis of SLC3A2 expression following expression of indicated shRNAs. **f** Experimental setup for CRISPR screens using p-RPS6 S240/244 as an endpoint. Details in methods sections. **g** FACS analysis of surface SLC3A2 (CD98) on MM lines expressing indicated control or RAS-targeted shRNAs. Representative data; n=3. **h** Immunofluorescence of LAMP1 (red) and SLC3A2 (green) in RPMI 8226 and SKMM1 cells expressing shCTRL or shSLC3A2.2. Representative images, n=3. Source data are provided as a Source Data file.

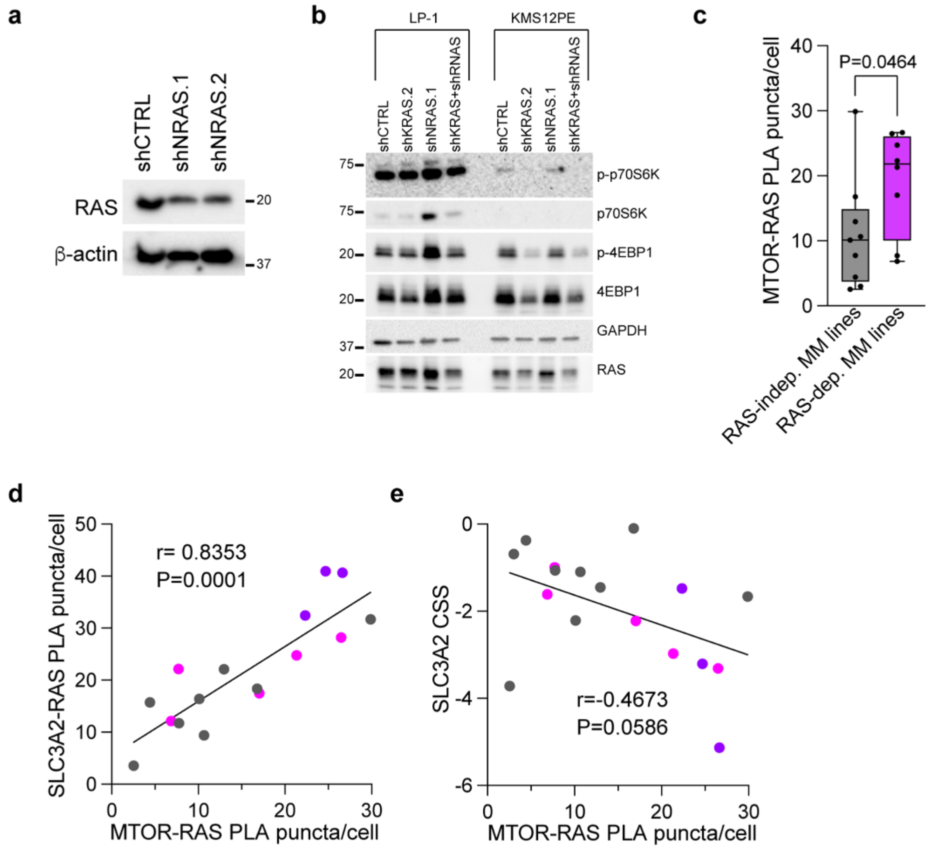

Supplemental Figure 4. RAS and MTOR associate in multiple myeloma cells. a Western blot analysis of NRAS knockdown in SKMM1 cells from Fig. 4c, n=1. **b** Western blot analysis of mTORC1 signaling outputs p-4EBP1 (S65) and p-p70S6K (T389) following knockdown of KRAS, NRAS or KRAS/NRAS in LP1 and KMS12PE cells expressing wild type RAS isoforms. Representative blots; n=4. **c** Average MTOR-RAS PLA puncta/cell for 17 MM cell lines. Each dot represents an individual MM line. P-value from one-tailed Mann-Whitney test; representative data, n=2. **d** Correlation between MTOR-RAS PLA (x-axis) and SLC3A2-RAS PLA (y-axis) in 16 MM cell lines. Spearman r value and two-tailed P value calculated by correlation analysis in Graphpad Prism. **e** Correlation between MTOR-RAS PLA (x-axis) and SLC3A2 CSS (y-axis) in 17 MM cell lines. Spearman r value and two-tailed P value calculated by correlation analysis in Graphpad Prism. Source data are provided as a Source Data file.

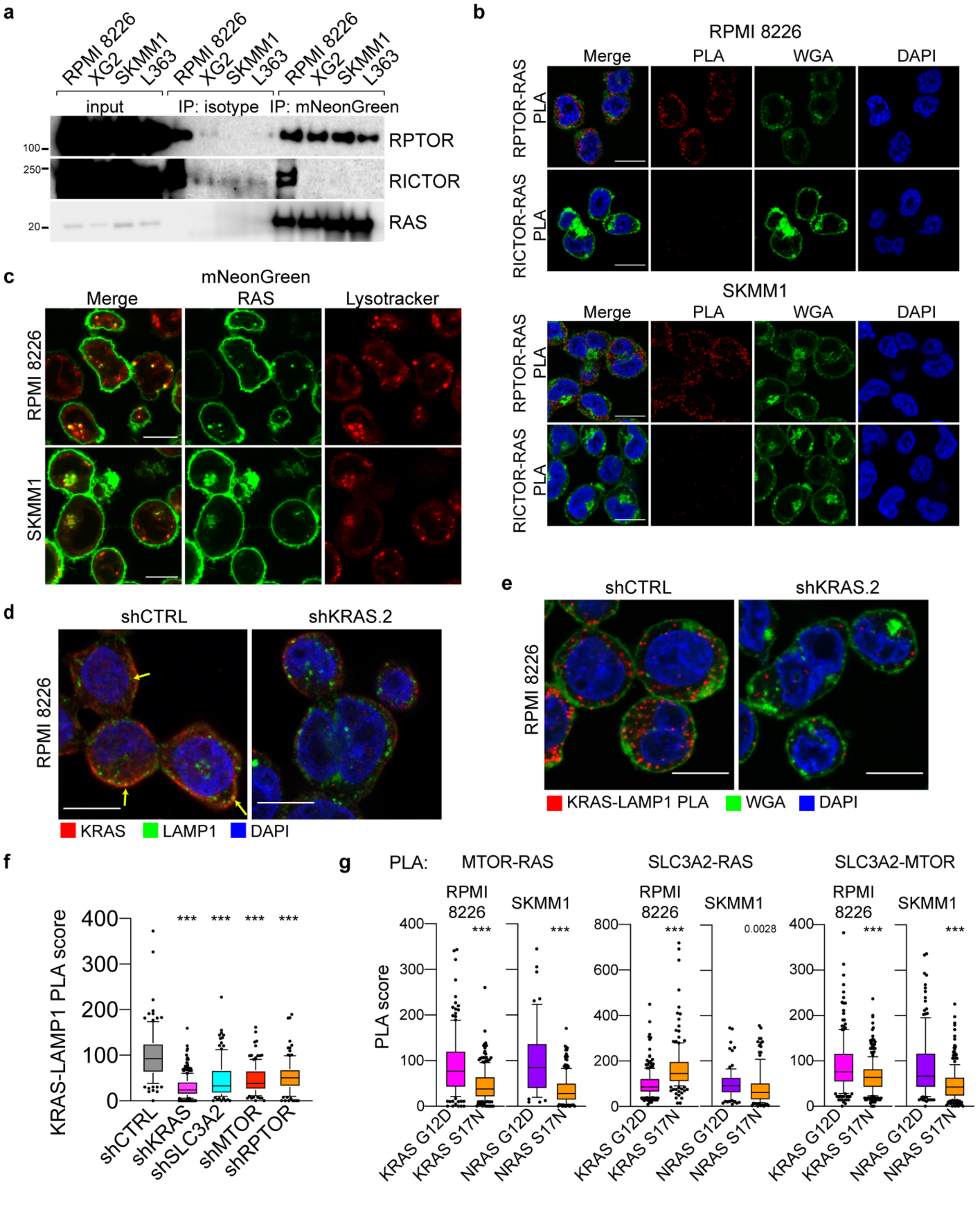

Supplemental Figure 5. RAS localizes to endolysosomes and engages RPTOR. a Co-immunoprecipitation of RPTOR and RICTOR with mutant isoforms of mNeonGreen-tagged KRAS and NRAS. mNeonGreen-tagged KRAS^{G12D} was used in RPMI 8226 and XG2, NRAS^{G12D} in

SKMM1 and NRAS^{Q61L} in L363. Representative blots; n=3. **b** Confocal images of RPTOR-RAS or RICTOR-RAS proximity ligation assay (PLA) in RPMI 8226 and SKMM1 cells with PLA (red), wheat germ agglutinin (WGA; green) and DAPI (blue) staining. Scale bar is 10 μm. Representative images; n=3. **c** Fluorescence imaging of live RPMI 8226 cells expressing mNeonGreen-KRAS^{G12D} or SKMM1 cells expressing mNeonGreen-NRAS^{G12D} in green with Lysotracker Red staining in red. Scale bar is 10 μm. Representative images; n=2. **d** Immunofluorescence of KRAS (red), LAMP1 (green) and DAPI (blue) in RPMI 8226 cells. Scale bar is 10 μm. Yellow arrows highlight areas of KRAS and LAMP1 overlap. Representative images, n=2. **e** Imaging of KRAS-LAMP1 PLA (red) in RPMI 8226 cells. Cells were costained with WGA (green) and DAPI (blue). Scale bar is 10 μm. Representative images, n=4. **f** Quantitation of KRAS-LAMP1 PLA in RPMI 8226 cells expressing control shRNAs or shRNAs targeting KRAS, SLC3A2, MTOR or RPTOR. Data pooled from 4 independent experiments; the number of cells quantified per condition listed in the source data file. *** denotes p-value <0.0001 by one-way ANOVA. Scale bar is 10 μm. **g** Quantitation of indicated PLAs in RPMI 8226 and SKMM1 cells expressing constitutively active (G12D) or dominant negative (S17N) versions of KRAS or NRAS. Data pooled from 3 independent experiments; the number of cells quantified per condition listed in the source data file. *** denotes p-value <0.0001 by unpaired two-tailed Mann Whitney t test. Source data are provided as a Source Data file.

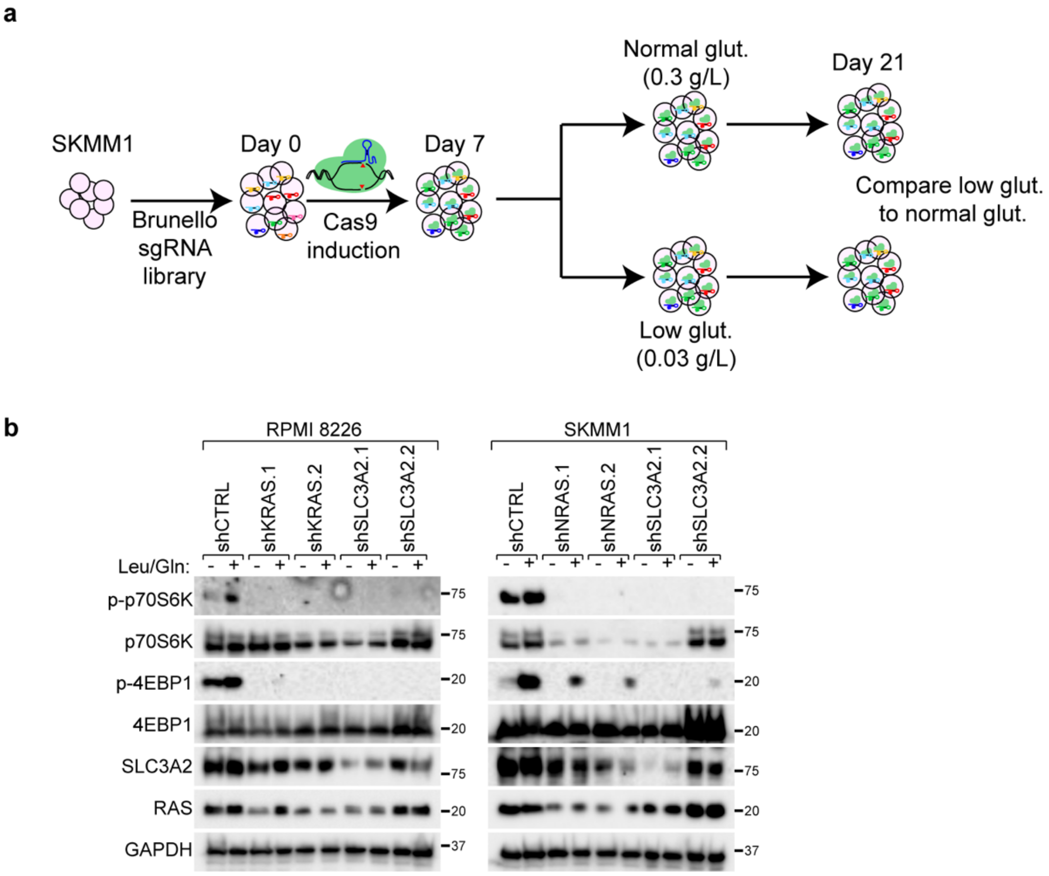

Supplemental Figure 6. Regulation of RAS-dependent mTORC1 signaling by amino acids. a Experimental setup for CRISPR modifier screen comparing SKMM1 cells grown under normal vs. glutamine restricted conditions. Details in Methods section. **b** Western blot analysis of mTORC1 signaling in RPMI 8226 (left) and SKMM1 (right) cells following amino acid starvation – or + provision of leucine and glutamine in cells expressing indicated shRNAs. Representative blots; n=4. Source data are provided as a Source Data file.

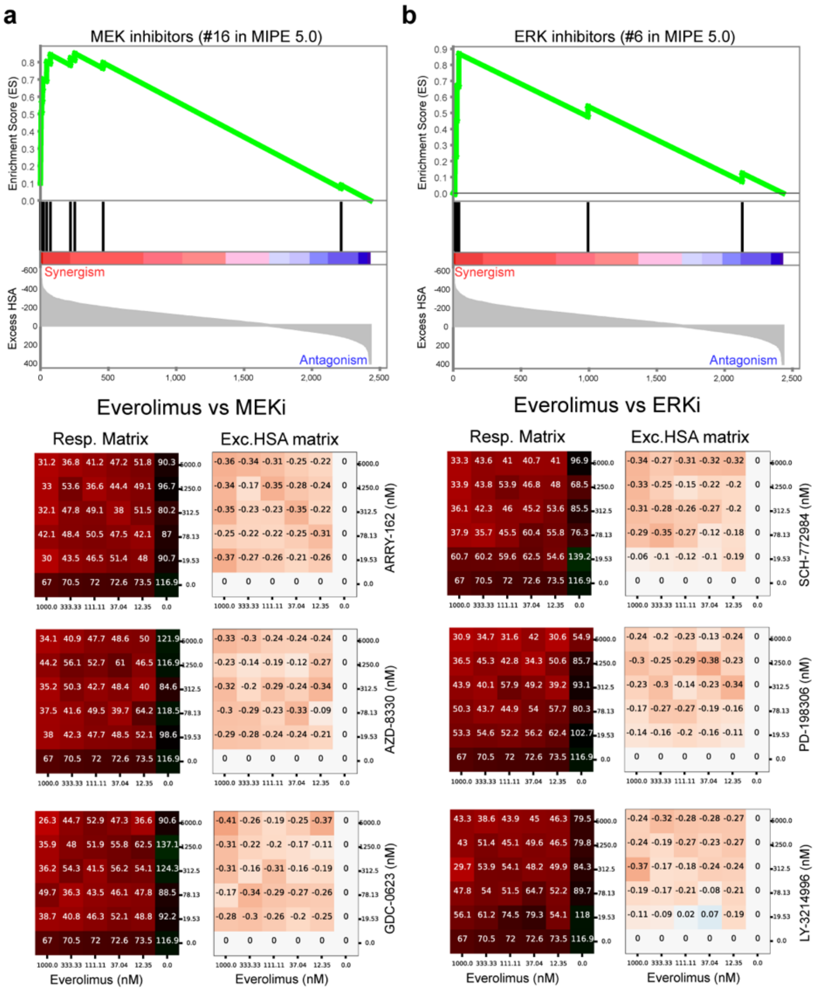

Supplemental Figure 7. A large-scale combinatorial drug screen reveals synergistic interaction and druggable vulnerabilities of RAS-dependent MM lines. Drug Set Enrichment Analysis (DSEA) of the Everolimus vs MIPE5.0 screen in SKMM1 and PRMI 8226. The average Excess HSA was used to pre-rank combinatorial outcomes before running DSEA. Enrichment plots for MEK (**a**) and ERK (**b**) inhibitors are shown, together with the 6x6 blocks for the 3 most-synergistic drugs within each target-class.

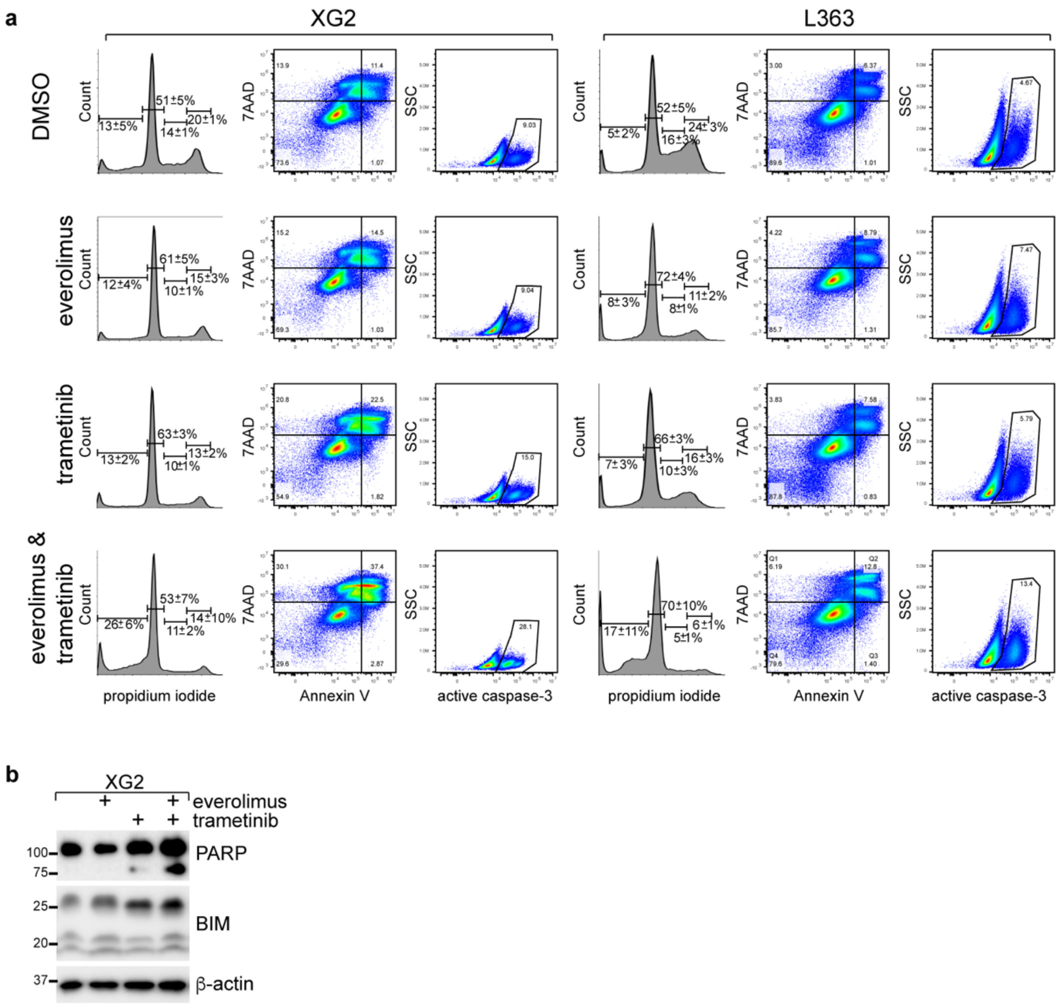

Supplemental Figure 8. Combinations of mTORC1 and MEK inhibitors induce apoptosis in MM cells. a Analysis of cell cycle and viability in XG2 (left) and L363 (right) MM cell lines treated with DMSO, 50 nM everolimus, 5 nM trametinib or both drugs. For propidium iodide cell cycle analysis, bars are labeled sub-G1, G1, S or G2 with indicated percentages and standard deviations. For 7AAD/Annexin V and cleaved caspase-3 staining percent of cells in indicated gates are listed in plots. Representative experiments, n=3. **b** Western blot analysis of BIM expression and PARP cleavage in XG2 cells treated with indicated drugs. Representative blots; n=2. Source data are provided as a Source Data file.
