# Supplementary material for: Oncogenic RAS commandeers amino acid sensing machinery to aberrantly activate mTORC1 in multiple myeloma
Source: Nat Commun. 2022 Sep 17;13:5469. doi: 10.1038/s41467-022-33142-x (PMC9482638; doi:10.1038/s41467-022-33142-x)
Supplement: Supplementary file 2 — Reporting Summary [file 41467_2022_33142_MOESM2_ESM.pdf]

nature portfolio

Corresponding author(s): Ryan M. Young

Last updated by author(s): Aug 12, 2022

Reporting Summary

Nature Portfolio wishes to improve the reproducibility of the work that we publish. This form provides structure for consistency and transparency in reporting. For further information on Nature Portfolio policies, see our Editorial Policies and the Editorial Policy Checklist.

Statistics

For all statistical analyses, confirm that the following items are present in the figure legend, table legend, main text, or Methods section.

n/a	Confirmed	
☐	☒	The exact sample size (*n*) for each experimental group/condition, given as a discrete number and unit of measurement
☐	☒	A statement on whether measurements were taken from distinct samples or whether the same sample was measured repeatedly
☐	☒	The statistical test(s) used AND whether they are one- or two-sided<br>*Only common tests should be described solely by name; describe more complex techniques in the Methods section.*
☐	☒	A description of all covariates tested
☐	☒	A description of any assumptions or corrections, such as tests of normality and adjustment for multiple comparisons
☐	☒	A full description of the statistical parameters including central tendency (e.g. means) or other basic estimates (e.g. regression coefficient) AND variation (e.g. standard deviation) or associated estimates of uncertainty (e.g. confidence intervals)
☐	☒	For null hypothesis testing, the test statistic (e.g. *F*, *t*, *r*) with confidence intervals, effect sizes, degrees of freedom and *P* value noted<br>*Give P values as exact values whenever suitable.*
☒	☐	For Bayesian analysis, information on the choice of priors and Markov chain Monte Carlo settings
☒	☐	For hierarchical and complex designs, identification of the appropriate level for tests and full reporting of outcomes
☐	☒	Estimates of effect sizes (e.g. Cohen's *d*, Pearson's *r*), indicating how they were calculated

Our web collection on statistics for biologists contains articles on many of the points above.

Software and code

Policy information about availability of computer code

Data collection	CRISPR screen reads were generated using NextSeq 1000/2000 Control Software (v. 1.2.036376) (Illumina) and demultiplexed using DRAGEN (v.3.7.4) (Illumina). Images were collected on Zeiss Zen Black (v2.1). FACS data was collected on BD Cellquest (v6.0). Western blot images were collected with Image Lab Touch Software (v2.3.0.07) (BioRad)
Data analysis	CRISPR screen reads were extracted from fastq files and normalized using custom perl scripts and Bowtie2 (version 2.2.9).<br>Correlation and statistical analysis of normalized sgRNA counts were performed with in house custom scripts and implemented in TIBCO Spotfire S+® 8.2 for Windows and R 64bit version 3.4.0.<br>FACS files were analyzed using FlowJo version 10.<br>Mass spec data was processed using MaxQuant version 1.6.0.1 linked to the UniProtKB/Swiss-Prot human database.<br>Data analysis was performed on Microsoft Excel version 16.63.1 and Prism version 10.<br>GSEA software (v4.0.3)

For manuscripts utilizing custom algorithms or software that are central to the research but not yet described in published literature, software must be made available to editors and reviewers. We strongly encourage code deposition in a community repository (e.g. GitHub). See the Nature Portfolio guidelines for submitting code & software for further information.

Data

Policy information about availability of data

All manuscripts must include a data availability statement. This statement should provide the following information, where applicable:

- Accession codes, unique identifiers, or web links for publicly available datasets
- A description of any restrictions on data availability
- For clinical datasets or third party data, please ensure that the statement adheres to our policy

Mass spectrometry data was uploaded to the Proteomics Identification Database (PRIDE) under accession number PXD031662. RNA seq data was uploaded to the Gene Expression Omnibus (GEO) under accession number GSE196231. The CRISPR screen score (CSS) data used in this study are provided in the Source Data file. Raw images available upon request. Public databases used herein can be found at https://www.uniprot.org/proteomes/UP000005640 and https://toppgene.cchmc.org. Source Data are provided with this paper.

Human research participants

Policy information about studies involving human research participants and Sex and Gender in Research.

Reporting on sex and gender	Of the 28 FFPE biopsy samples we obtained, only 8 samples had a recorded sex. Of these, 7/8 were listed as male. The remaining 20 samples had no recorded sex. Sex was not considered in the design of this study and we performed no sex- or gender based analyses with this data.
Population characteristics	This patient cohort had known KRAS and NRAS mutational status from targeted sequencing done previously for diagnostic reasons. Within this group of 28 MM biopsy samples, 11 expressed mutant KRAS, 4 expressed mutant NRAS and 13 were wild type for both KRAS and NRAS. Details in Source Data file.
Recruitment	All human samples were archival tissue submitted for consultation to the Department of Laboratory Medicine at the NIH Clinical Center. All samples were subsequently de-identified for PLA assays. Participants were not compensated.
Ethics oversight	Institutional Review Board approved protocols from the National Cancer Institute National Institutes of Health Protocol Review Office

Note that full information on the approval of the study protocol must also be provided in the manuscript.

Field-specific reporting

Please select the one below that is the best fit for your research. If you are not sure, read the appropriate sections before making your selection.

[x] Life sciences [ ] Behavioural & social sciences [ ] Ecological, evolutionary & environmental sciences

For a reference copy of the document with all sections, see nature.com/documents/nr-reporting-summary-flat.pdf

Life sciences study design

All studies must disclose on these points even when the disclosure is negative.

Sample size	Sample size was limited by constraints of the experimental systems, but were estimated to be large enough to obtain reproducible results.
Data exclusions	For CRISPR screen data, only the most correlated sgRNAs were used to generate CSS (see methods). Mass spec data was filtered for frequently observed contaminants via the Andromeda search engine and low confidence uniprot peptides (see methods). Mouse deaths resulting from oral gavage were excluded. Data was excluded if positive or negative control samples failed.
Replication	All experiments were reproduced independently, unless otherwise stated. The number of biologically independent replicates is listed in the figure legend and/or source data for each experiment.
Randomization	Cell lines were not randomized as analysis of RAS-dependent features required knowledge of RAS dependency and sample genotype. MM patients were grouped by RAS mutational status. For imaging, fields of cells were randomly chosen. For biochemical analysis, all cells within samples were analyzed.
Blinding	Investigators were generally not blinded as the experimental conditions required investigators to know the identity of the samples. The one exception was data provided in Fig. 6A-B, where the mutational status of samples was not revealed till after analysis.

Reporting for specific materials, systems and methods

We require information from authors about some types of materials, experimental systems and methods used in many studies. Here, indicate whether each material, system or method listed is relevant to your study. If you are not sure if a list item applies to your research, read the appropriate section before selecting a response.

Materials & experimental systems

n/a	Involved in the study
☐	☒ Antibodies
☐	☒ Eukaryotic cell lines
☒	☐ Palaeontology and archaeology
☐	☒ Animals and other organisms
☒	☐ Clinical data
☒	☐ Dual use research of concern

Methods

n/a	Involved in the study
☒	☐ ChIP-seq
☐	☒ Flow cytometry
☒	☐ MRI-based neuroimaging

Antibodies

Antibodies used

Antibody Species Clone Supplier Catalog No. Lot No.
anti-pan RAS mouse C-4 SCBT sc-166691 J0120
anti-mTOR mouse 6H9B10 Biolegend 659202 B241067
anti-LAMP1 mouse H4A3 SCBT sc20011 D1612
anti-CD98 (SLC3A2) rabbit BETHYL A304-331A
anti-mTOR rabbit 7C10 CST 2983 16, 19
anti-p-4E-BP1 (S65) rabbit CST 4165 16
anti-p-p70S6K (T389) rabbit 108D2 CST 9234 12
anti-4E-BP1 rabbit 53H11 CST 9644 12
anti-p70S6K rabbit CST 9202 20
anti-MEK1/2 rabbit D1A5 CST 8727 5
anti-p-MEK1/2 (S217/221) rabbit 41G9 CST 9154 18
anti-p-PKCa/b (T638/641) rabbit CST 9375 4
anti-PKCa rabbit D7E6E CST 59754 1
anti-CD98 (SLC3A2) rabbit D3F9D CST 47213 1
anti-GAPDH mouse 0411 SCBT sc-47724 E2219
anti-b-actin goat C-11 SCBT sc-1615 E2314
anti-CD138 Alexa 647 mouse MI15 Biolegend 356524 B271942
anti-active Caspase 3 APC rabbit BD 51-68655X 8024887
anti-CD54 APC mouse HCD54 Biolegend 322712 B263252
anti-CD98 (SLC3A2) mouse 4F2 SCBT sc-59145 E2314
anti-BioID2 mouse Novus nbp2-59941 CRT/17/86
anti-mouse Alexa 647 goat CST 4410 10
anti-rabbit Alexa 488 goat CST 4412 18
anti-KRAS mouse F234 SCBT sc-30 B0422
anti-NRAS mouse F155 SCBT sc-31 B1517
anti-p-RPS6 (S240/244) Pac. Blue rabbit D68F8 CST 5018S 1
anti-RPS6 Alexa 488 rabbit 54D2 CST 5317S 9
anti-BIM rabbit C34C5 CST 2933S 13
anti-PARP rabbit CST 9542 3

Validation

All Antibodies were validated by the manufacturer. In addition, we validated that all antibodies showed the expected phenotype for a given assay. For almost all antibodies, we validated loss of antibody detection of protein following shRNA-mediated knockdown of protein levels. This was done by either western blot analysis, FACS or confocal microscopy. When we did not validate specificity by knockdown, as was the case for certain antibodies used for western blot analysis, we verified that the antibody yielded the expected molecular weight and banding pattern.

anti-pan RAS mouse C-4 SCBT sc-166691 We validated that the RAS band at ~21 kDa decreased upon KRAS or NRAS knockdown by western blot
anti-mTOR mouse 6H9B10 Biolegend 659202 We validated that PLAs including this antibody had decreased signal upon MTOR knockdown
anti-LAMP1 mouse H4A3 SCBT sc20011 We validated that PLAs including this antibody had decreased signal upon LAMP1 knockdown
anti-CD98 (SLC3A2) rabbit BETHYL A304-331A We validated that PLAs including this antibody had decreased signal upon SLC3A2 knockdown
anti-mTOR rabbit 7C10 CST 2983 We validated that PLAs including this antibody had decreased signal upon MTOR knockdown
anti-p-4E-BP1 (S65) rabbit CST 4165 We validated bands at the correct MW decreased upon mTORC1 inhibition by western blot
anti-p-p70S6K (T389) rabbit 108D2 CST 9234 We validated bands at the correct MW decreased upon mTORC1 inhibition by western blot
anti-4E-BP1 rabbit 53H11 CST 9644 From CST: 4E-BP1 (53H11) Rabbit mAb detects endogenous levels of total 4E-BP1 protein
anti-p70S6K rabbit CST 9202 From CST: p70 S6 Kinase Antibody detects endogenous levels of total p70 S6 kinase protein. This antibody also recognizes p85 S6 kinase
anti-MEK1/2 rabbit D1A5 CST 8727 From CST: MEK1/2 (D1A5) Rabbit mAb recognizes endogenous levels of total MEK1 and MEK2

proteins. This antibody is predicted to cross-react with MEK1/MEK2 orthologs in a variety of species.
anti-p-MEK1/2 (S217/221) rabbit 41G9 CST 9154 We validated bands at the correct MW decreased upon KRAS and NRAS knockdown by western blot
anti-p-PKCa/b (T638/641) rabbit CST 9375 From CST: Phospho-PKC alpha/beta II (Thr638/641) Antibody detects PKC alpha only when phosphorylated at threonine 638 and PKC beta II only when phosphorylated at threonine 641. This antibody also reacts with gamma.
anti-PKCa rabbit D7E6E CST 59754 From CST: Monoclonal antibody is produced by immunizing animals with a synthetic peptide corresponding to residues surrounding Val664 of human PKCα protein.
anti-CD98 (SLC3A2) rabbit D3F9D CST 47213 We validated bands at the correct MW decreased upon SLC3A2 knockdown by western blot
anti-GAPDH mouse O411 SCBT sc-47724 We observed a single band at the correct molecular weight by western blot
anti-b-actin goat C-11 SCBT sc-1615 We observed a single band at the correct molecular weight by western blot
anti-CD138 Alexa 647 mouse MI15 Biolegend 356524 We validated by FACS that CD138 expression decreased upon CRISPR knockout of CD138
anti-active Caspase 3 APC rabbit BD 51-68655X We validated that staining by this antibody increased with etoposide treatment of cells to induce apoptosis
anti-CD54 APC mouse HCD54 Biolegend 322712 We validated that CD54 expression decreased by FACS after CD54 CRISPR knockout
anti-CD98 (SLC3A2) mouse 4F2 SCBT sc-59145 We validated that SLC3A2 expression decreased by FACS with SLC3A2 knockdown
anti-mouse Alexa 647 goat CST 4410 From CST: F(ab')2 fragments are prepared from goat antibodies that have been adsorbed against human IgG and human serum.

Eukaryotic cell lines

Policy information about cell lines and Sex and Gender in Research

Cell line source(s)

ARP1C Human multiple myeloma cell line NCI
EJM Human multiple myeloma cell line DMSZ ACC 560
H1112 Human multiple myeloma cell line NCI
INA6 Human multiple myeloma cell line DMSZ ACC 862
JJN3 Human multiple myeloma cell line DMSZ ACC 541
JK6L Human multiple myeloma cell line DMSZ ACC 860
KSM12PE Human multiple myeloma cell line JCRB JCRB0430
KMS26 Human multiple myeloma cell line JCRB JCRB1187
KMS34 Human multiple myeloma cell line JCRB JCRB1195
L363 Human multiple myeloma cell line DMSZ ACC 49
LP1 Human multiple myeloma cell line DMSZ ACC 41
MM.1.144 Human multiple myeloma cell line NCI
OCI-MY5 Human multiple myeloma cell line NCI
RPMI 8226 Human multiple myeloma cell line ATCC CCL-155
SKMM1 Human multiple myeloma cell line NCI
XG2 Human multiple myeloma cell line NCI
XG6 Human multiple myeloma cell line NCI
NCI-H1299 Human lung adenocarcinoma cell line ATCC CRL-5803
NCI-H2122 Human lung adenocarcinoma cell line ATCC CRL-5985
A549 Human lung adenocarcinoma cell line ATCC CCL-185
LS180 Human colon adenocarcinoma cell line ATCC CL-187
SW837 Human colon adenocarcinoma cell line ATCC CCL-235
GP2D Human colon adenocarcinoma cell line Sigma 95090714
LOVO Human colon adenocarcinoma cell line ATCC CCL-229
SW1463 Human colon adenocarcinoma cell line ATCC CCL-234
SK-CO-1 Human colon adenocarcinoma cell line ATCC HTB-39
LS513 Human colon adenocarcinoma cell line ATCC CRL-2134
HCT8 Human colon adenocarcinoma cell line ATCC CCL-224
ASPC1 Human pancreatic adenocarcinoma cell line ATCC CRL-1682
PANC1 Human pancreatic adenocarcinoma cell line ATCC CRL-1469
293T Transformed Human kidney cell line ATCC CRL-3216
293FT Transformed Human kidney cell line Thermo Fisher R70007

Authentication

We used a “DNA fingerprinting” method to test for the presence or absence of 16 common copy number variants allowing the detection of cross-contamination. Using multiplex PCR this strategy generates binary fingerprints that can were used compare pre- and post-single cell cloning of Cas9 variants.

Mycoplasma contamination

All cell line tested negative for mycoplasma using using the MycoAlert Mycoplasma Detection Kit (Lonza). Cell lines were tested regularly and preventative treatment was undertaken using MycoZap (Lonza) and Plasmocin (InvivoGen).

Commonly misidentified lines
(See ICLAC register)

No cell lines used in this study among commonly misidentified lines.

Animals and other research organisms

Policy information about studies involving animals; ARRIVE guidelines recommended for reporting animal research, and Sex and Gender in Research

Laboratory animals	All mouse experiments were approved by the National Cancer Institute Animal Care and Use Committee (NCI-ACUC) and were performed in accordance with NCI-ACUC guidelines and under approved protocols. Female NSG (non-obese diabetic/severe combined immunodeficient/common gamma chain deficient) mice were obtained from NCI Fredrick Biological Testing Branch and used for the xenograft experiments between 6-8 weeks of age. Mice were housed in specific pathogen-free facility in ventilated microisolator cages with 12 hour light and 12 hour dark cycles at 72F and 40-60% relative humidity.
Wild animals	This study did not utilize wild animals.
Reporting on sex	All experiments were performed in female NSG mice, as reported in the methods.
Field-collected samples	This study did not utilize field-collected samples.
Ethics oversight	National Cancer Institute Animal Care and Use Committee (NCI-ACUC)

Note that full information on the approval of the study protocol must also be provided in the manuscript.

Flow Cytometry

Plots

Confirm that:

- [x] The axis labels state the marker and fluorochrome used (e.g. CD4-FITC).
- [x] The axis scales are clearly visible. Include numbers along axes only for bottom left plot of group (a 'group' is an analysis of identical markers).
- [x] All plots are contour plots with outliers or pseudocolor plots.
- [x] A numerical value for number of cells or percentage (with statistics) is provided.

Methodology

Sample preparation	MM cell lines were transduced with sgCD54 vector co-expressing GFP and stained with anti-CD54 (1:1000) to select Cas9-expressing cells as previously described 2. SLC3A2 surface expression was measured by staining 2x105 cells on ice with anti-CD98 (1:500) for 20 minutes in FACS buffer (PBS with 2% BSA). Cells were washed with FACS buffer and stained with anti-mouse-Alexa647 (1:1000; CST) for 20 minutes on ice, then washed again and resuspended in 250l of FACS buffer.
Instrument	BD FACS Calibur and Beckman Coulter CytoFlex LX
Software	Data was collected with CellQuest Pro and analyzed with FlowJo v10 (Treestar).
Cell population abundance	At least 10,000 events were collected and analyzed.
Gating strategy	Live cells were gated using FSC/SSC parameters. For shRNA experiments GFP positive cells were gated using untransduced GFP negative controls.

- [x] Tick this box to confirm that a figure exemplifying the gating strategy is provided in the Supplementary Information.
